# Supplementary material for: Periodicity in Steller’s eider (Polysticta stelleri) population size and density on the Arctic Coastal Plain, Alaska, revealed using generalized additive models
Source: PLoS One. 2026 Apr 30;21(4):e0347122. doi: 10.1371/journal.pone.0347122 (PMC13132232; doi:10.1371/journal.pone.0347122)
Supplement: S1 Table — This is an unpublished USFWS report that describes the field experiment and analysis to estimate detection probability of Steller’s eider by an aerial observer in 2017 and 2018. Table 3 is used to calculate the detection probability averaged over distance. (DOCX) [file pone.0347122.s001.docx]

**Estimating detection probability in the Steller’s eider Barrow Triangle aerial survey using a double-observer sightability model**

**2018 Report**

To: Neesha Stellrecht, ES Recovery Program Branch Chief

From: Catherine Bradley, FES Biometrician

CC: Debora Nigro, BLM; Tim Obritschkewitsch, ABR; Nathan Graff, FWS; Kate Martin, FWS

**Background**

The Barrow Triangle Steller’s eider aerial survey has been flown annually since 1999. Counts of single males, pairs, and flocks of Steller’s eiders are reported each year (e.g. Obritschkewitsch and Ritchie 2017); however, a robust estimate of the detection probability associated with this survey is needed if population estimates are to be produced from the data. In 2017 and 2018, we implemented a protocol using decoys and double-observers to estimate the unconditional detection probability of Steller’s eiders.

Double-observers (DO models) are frequently used in aerial surveys to estimate detection probabilities and associated population sizes (Pollock and Kendall 1987). However, double-observer surveys can result in positively-biased detection probabilities as they only include individuals observed by at least one surveyor (i.e., conditional detection; Barker 2008). This bias generates an underestimation of the population size.

Sightability surveys (S models) use marked animals – most frequently those previously radio-tagged – to obtain an unconditional estimate of detection probability (unconditional in the sense that an observation by at least one surveyor is not required for a marked animal to be included in the dataset; Steinhorst and Samuel 1989). Sighting covariates are collected for both detected and undetected individuals, providing a model of the factors contributing to an unconditional detection estimate. Because sightability trials often depend on secondary marks like radio tags that can be difficult and expensive to deploy, the precision of the resulting detection probabilities can be poor due to small sample sizes.

Griffin et al. (2013) developed a hybrid modeling framework to leverage the strengths of both the double-observer and sightability methods (DO-S models). A key assumption to the DO-S model, and to all sightability models, is that tagged animals are comparable to untagged animals with respect to sightability. The current study discussed here adapted the Griffin et al. (2013) design by using painted duck decoys as the marked animals. Decoys have been used in other aerial surveys of birds to correct counts for unconditional detection (Pearse et al. 2008) or to evaluate the visibility biases in existing aerial surveys (Strobel and Butler 2015). A critical assumption in these models is that the detection process for decoys is equivalent to that of live birds. This can, at best, only apply to birds not in flight.

In addition to the primary goal of this study, surveyors collected double-observer data for king and spectacled eiders. This data is analyzed here in conjunction with sightability covariates and estimated conditional detection probabilities are reported.

This report compiles and analyzes the data obtained in 2017 and 2018. Modifications were made to data processing and the analysis. As a result, this report supersedes the interim report made after the 2017 field season.

**Decoy Deployment and the Flight**

Each year, male Steller’s eider decoys (N=40 in 2017; N=37 in 2018) were placed in flight transects in the northernmost portion of the study area. These transects are the most easily accessible to the ground crew and represent the area where the majority of the Steller’s eiders have been observed during the aerial survey (Obritschkewitsch and Ritchie 2017). In 2017, most decoys (N=37) were placed in decoy transects that were between standard survey transects and, thus, not part of the standard survey. After confirming that waypoint locations were accurate enough to distinguish between live birds and decoys, and with concerns that surveyor behavior may differ on dedicated decoy transects, decoys were placed on transects that were included in the standard survey in 2018.

Using information from the ground survey effort, decoys in 2017 were placed in different habitat types (*Arctophila*-dominant shallow and deep ponds, *Carex*-dominant shallow and deep ponds, deep open ponds, streams, dry tundra, flooded tundra, and ditches) in relative proportion to live bird observations made over several years. These habitat classifications were simplified to shallow pond, deep pond, stream, and tundra for both deployment and observation in 2018.

Under the constraints of available habitat, decoys were distributed at various distances up to 200m from the transect center and distributed north and south of the transect line. Transects with decoys deployed on them were flown in both directions (east and west) so that all decoys were available to the right side of the plane. Full deployment data for the decoys can be found in Appendix A1.

The Barrow Triangle aerial survey was flown June 20-23, 2017 and June 25-30, 2018. Two surveyors, alternating between the front and rear seat, sat on the right side conducting the double-observer sightability trials. A third surveyor was stationed on the left side of plane behind the pilot so that survey counts could be comparable to previous years. Details about the flight protocols can be found in Obritschkewitsch and Ritchie (2017).

Measures were taken to try to ensure observations were made independently by the front and rear surveyor. On-the-fly reconciliation was performed to match waypoints, but surveyors recorded habitat and distance bin individually. In addition to these two covariates, transect-specific covariates (front seat surveyor, flight direction, sun angle relative to the nose of the airplane, ground speed, and cloud cover) were also recorded.

**The Data**

*2017*

Fifteen decoys (37.5%) were observed by at least one surveyor and five were seen by both. Several decoys were noted as unusual by the surveyors (e.g., “too white” or “riding high in the water”). However, one of the three decoys placed in a standard transect was observed by one surveyor and marked as a live bird. This was changed to a decoy observation post-season because the recorded waypoint location and agreement in covariate values (distance and habitat) indicated it was highly likely to be the decoy. No decoys placed on dry tundra or in ditches were seen by the surveyors. Nearly half of the decoys seen by surveyors were in the closest distance bin. Three decoys were observed in the most distant bin. These three were seen by surveyor TO (once as front seat surveyor, twice as rear seat surveyor).

In addition to the decoy observations, 14 observations of male Steller’s eiders were also made by the surveyors. Two of these observations were of two males in close proximity to one another; these male pairs were each treated as a single sighting (i.e., no group size effect). Four of the male Steller’s eiders were seen by both surveyors (28.6%). Four were in flight and these were removed from the analysis since we have no method to calculate an unconditional detection probability for birds in flight.

Figure 1 shows the detections of decoy and live birds in relation to flight transects in 2017.

There was a good deal of disagreement by the surveyors in calling habitat type at the level of detail set out in the protocols (e.g., shallow *Arctophila*-dominated pond versus shallow *Carex*-dominated pond). Where true habitat type was known (decoy deployments), there was also disagreement between the surveyors and truth. However, a broader categorization was consistently agreed upon and in concordance with known truth: shallow water, deep water, dry tundra, and stream.

A miscommunication resulted in different distance bins used by the decoy deployment team (20-80m, 80-130m, and 130-190m) and the aerial survey team (0-50m, 51-100m, 101-150m, 151-200m). However, reconciliation between the datasets resulted in three distance bins (those used by the decoy deployment team) that suggested good agreement between truth (decoy deployments) and surveyor data (12 of 15 correctly classified). This covariate was included in the model as a factor.

*2018*

Decoys were placed in transects that were part of the standard survey in 2018. As in 2017, surveyors attempted to distinguish between decoys and live birds. Based on waypoint locations, two live observations were changed to decoys and five observations called decoys in the field were changed to live observations. We made the decision to call the latter live observations, rather than census them from the data as possible false positives, because surveyors had other options when unsure about the species identity of a bird (unknown or unknown eider). These reclassifications occurred at all distance bins and all habitat types. We assume that because surveyors knew there were a number of decoys placed in the northernmost transects they may have defaulted to calling observations decoys if deemed at all reasonable. This is an unavoidable consequence of decoys greatly outnumbering live birds that would be difficult to attend to in survey design, but can be reasonably addressed post-survey if waypoint locations are accurately recorded.

Fourteen decoys (37.8%) were seen by at least one surveyor; ten were seen by both. No observations of decoys placed in the furthest distance bin (150-200m) were made (N= 7). The simplified habitat classifications (shallow pond, deep pond, stream, ground) resulted in good agreement between surveyors and between surveyors and truth. Three observations of males in flight, including one where a pair of males were seen, were removed from the dataset for analysis.

Figure 2 shows the detections of decoy and live birds in relation to flight transects in 2018.

We used the four distance bins used by the surveyors in 2017 for decoy deployment and survey observations. For the purposes of modeling, true distance was used for decoys, agreed distance was used for live birds seen by both surveyors, the front surveyor’s distance was used if there was disagreement, and, if a bird was only seen by one surveyor, their estimated distance was used.

The data for this analysis is given in Appendix A2.

**Analysis and Results**

Because distance was binned differently between years (3 bins in 2017 and 4 bins in 2018), yearly data was analyzed separately. In addition to habitat type and distance, front surveyor, transect direction (east or west) and sun degree were considered in candidate models. Sun degree is the direction from which sunlight hit the plane during the transect flight where 0 is due north and 180 is due south.

Huggins type mark-recapture models (1989, 1991) were fit in a Bayesian framework using R (vers. 3.5.0; R Core Team 2018) with R2OpenBUGS (vers. 3.2; Sturtz et al. 2005). With small datasets such as these, there is a risk of overfitting a model. To attempt to accommodate this, model building was performed in two phases. First, a model allowing for different detection rates between front and rear surveyors was compared to model without a seat difference. From the simplest, best fitting, model of these two as determined by DIC, models including type (decoy or live observation) and one (or two, in the case of sun degree and transect direction) additional sighting covariate were constructed. The type variable allows for the comparison of unconditional and conditional detection probabilities. Final model selection was made using DIC and, when no single model held more than 80% of the model weight, model averaging was employed for inference. Model convergence, following 10,000 iterations, 2,000 burn-in rate, and using 3 chains, was assessed by traceplots and the R-hat statistic (Brooks and Gelman 1998).

Reported detection estimates are given for the front seat surveyor only. 95% confidence intervals were calculated using the posterior MCMC draws retained from the model fit. Where model averaging was required, a weighted average of estimated detection, $\hat{p_{w}}$, was calculated for all *k* included models.

$$\hat{p_{w}}= \sum_{k=1}^{K} \hat{p_{k}}w_{k}$$

Again, 95% confidence intervals for these weighted detection probabilities were estimated using the posterior MCMC draws retained from the model fits.

*2017*

There was no evidence to support an effect of surveyor position (equal: DIC=122.7; unequal: DIC=124.6). Three models, those including sun angle as a function of transect direction (33.2%), distance (28.6%), and no additional sighting covariates (24.6%), comprised a model set representing more than 80% of the total model weight and were used for inference by model averaging.

Model results suggest that unconditional detection declines from the near-distance bin (20-80m; 27.7% (95% CI: 20.7% - 35.5%) to the far-distance bin (130-190m; 20.1% (95% CI: 14.2% - 27.4%), but evidence of a strong trend is weak. Table 1 summarizes the relationship between distance and detection for the model set.

Table 1. Estimated detection probabilities as a function of distance for the live birds (conditional on one observation) and decoys (unconditional) in 2017. Estimates are from model averaging three competing models of sun angle effect, distance, and constant detection. 95% confidence intervals are given in parentheses.

| Distance (m) | Conditional | Unconditional |
| --- | --- | --- |
| 1 (20-80) | 0.541 (0.395 – 0.682) | 0.277 (0.207 – 0.355) |
| 2 (80-130) | 0.521 (0.362 – 0.678) | 0.260 (0.188 – 0.343) |
| 3 (130-290) | 0.436 (0.289 – 0.601) | 0.201 (0.142 – 0.274) |

Note that the difference between conditional and unconditional detection estimates is of similar magnitude to the unconditional estimates themselves, suggesting a relative bias of approximately 100%.

Detection probabilities were higher when flying west (32.7% versus 27.1%), but the deleterious effect of the sun in the surveyors’ eyes was nearly five times greater when flying west as compared to flying east (Table 2).

Table 2. Estimated detection probabilities as a function of flight direction and sun angle (the direction from which sunlight hit the plane during the transect flight where 0 is due north and 180 is due south) for live birds (conditional on one observation) and decoys (unconditional) in 2017. Estimates are from model averaging three competing models of sun angle effect, distance, and constant detection. 95% confidence intervals are given in parentheses.

| Direction and Sun Angle | Conditional | Unconditional |
| --- | --- | --- |
| West 225 | 0.552 (0.413 – 0.684) | 0.286 (0.210 – 0.370) |
| West 180 | 0.598 (0.458 – 0.722) | 0.327 (0.241 – 0.422) |
| East 180 | 0.532 (0.387 – 0.722) | 0.271 (0.200 – 0.349) |
| East 135 | 0.521 (0.370 – 0.674) | 0.263 (0.189 – 0.352) |
|  |  |  |

*2018*

Similar to 2017, there was no support for an effect of surveyor position (equal: DIC=126; unequal: DIC=125.6) and so the more parsimonious, no seat effect, model was used to explore other sightability covariates. Among the set of candidate models, the model that included distance accounted for greater than 90% of the model weight and no other candidate model was supported by more than 3% model weight.

For both live (conditional) and decoy (unconditional) observations, detection in distances 0-50m and 50-100m were similar to one another and higher than detection at further distances. The difference in detection between conditional and unconditional observations was, on average, 38-46% among the distance bins. Table 3 summarizes these results.

Table 3. Estimated detection probabilities as a function of distance for the live birds (conditional on one observation) and decoys (unconditional) in 2018. 95% confidence intervals are given in parentheses.

| Distance (m) | Conditional | Unconditional |
| --- | --- | --- |
| 1 (0-50) | 0.941 (0.723 – 0.981) | 0.514 (0.338 – 0.689) |
| 2 (50-100) | 0.894 (0.646 – 0.980) | 0.457 (0.217 – 0.717) |
| 3 (100-150) | 0.623 (0.306 – 0.870) | 0.143 (0.048 – 0.306) |
| 4 (150-200) | 0.560 (0.212 – 0.859) | 0.114 (0.026 – 0.310) |

**Discussion**

This study is unique in two ways. First, most aerial surveys consist of a two-part process: observing a group of animals followed by counting the animals in the group. Each event in the process is susceptible to error. The Steller’s eider survey is timed to coincide with pairing and dispersion and, consequently, pairs are most often seen in discrete units. Therefore, error resulting from group counts can largely be ignored. Across both years, there were three occasions where two males were seen together. In two instances, these groups were treated as a single observation. In the third, the group was in flight and removed from the analysis.

It is possible that the presence of a female with a male has a group effect and makes the male more detectable. Though females are more visually inconspicuous, there presence, perhaps by movement, may draw a surveyor’s attention. Here we did not consider females in the detection process primarily because there can be considerable inconsistencies in two surveyors seeing a female in proximity to a male. Further, it has been a standard assumption that at the time of the survey a male is in a pair group, whether the female is seen or not.

A second unique characteristic to this study is that the number of marked animals (decoys) in the field exceeds the number of observed unmarked animals (live birds). Indeed, the rationale for the DO-S framework proposed by Griffith et al. (2013) was to improve upon the precision of detection estimates obtained from the small sample of marked animals typical of sightability studies. In the present study, live observations may play a different role in that they could allow for an assessment of the comparability between decoy detection and live bird detection. Such an assessment would require more years of data to build a larger set of live bird observations, but could be very informative to this and other studies.

Numerically dominant decoys could inadvertently impact the search patterns used by the surveyors, potentially introducing false positives into the dataset. In this study, surveyors know that there are a relatively large number of decoys in the northernmost transects. Data reconciliation in 2018 lead to the reclassification of seven observations – two live birds changed to decoys and five decoys changed to live birds. This appears reasonable as long as waypoints are accurately recorded and surveyors have alternative designations to use (e.g. unknown) if they are uncertain about an observation.

Despite these concerns, results suggest that the DO-S study design could be simplified to a sightability survey which does not require a second, backseat, observer on the same side of the plane. The discrepancy between conditional and unconditional detection estimates and indications that some classes of live birds have very low detection (e.g., those on dry tundra) indicate that decoy deployments are an important tool to assess detection in this sparse population. This would require continuing to flying those transects with decoys in both directions or increasing the number of decoys deployed so that surveyors on each side of the plane had sufficient opportunities to observe decoys.

Finally, an important result from this study is the indication that distance from the plane is a key covariate for detection. The four distance bin categories used in 2018 may have contributed to its stronger predictive value over the three used in 2017; however, the misclassification rate in 2018 was higher. Two alternatives to address this are reverting to the three categories used in 2017 and/or using distance markers on the struts or window of the plane to facilitate distance calls.

One important application of the Barrow Triangle Steller’s eider aerial survey is to document the spatial use of breeding Steller’s eiders throughout the study area. This study investigated means to estimate a detection probability for the survey so that a population estimate, rather than count index, could also be made. A broader issue to consider is which population would be estimated with a detection correction. As stated above, the survey is timed to coincide with pairing and dispersal throughout the breeding area defined by the Barrow Triangle study area. It is currently hypothesized that female adults may have high breeding site fidelity but that there is also annually variable breeding propensity (that proportion of the population that attempts breeding in any given year). Without an estimate of annual breeding propensity, an aerial survey count corrected for detection would best describe the proportion of the Barrow Triangle population that attempts breeding in a given year. It would be important to consider the length of the time series necessary to characterize trend in this data and what could be inferred about the Barrow Triangle population, or greater Alaska-breeding population, using a trend in the observed population.

**Spectacled Eider**

*Data*

Across 2017 and 2018, 280 unique observations of male spectacled eider groups were used to assess the detection probability of this species. Estimated detection probabilities are conditional on at least one surveyor observing a bird and are, therefore, likely positively biased. 131 were observed by only one surveyor and the remainder was observed by both. The majority of the observations were singles (N = 246), 52 males were seen in a pair, and eight groups of three were seen. Because few groups of three were observed, group size was dichotomized to one or more than one male. Unlike the double-observer study conducted on the Alaska Coastal Plain (Wilson et al. *in prep*), we did not include female spectacled eiders in the group size because there were discrepancies between surveyors in sighting female birds in proximity to males.

65 birds were observed in waterbodies in 2017 and equal numbers (N=40) were observed flying or flushing. This contrasts with 2018 when only 27 birds were observed in water, nearly one-half of all observations were of birds in flight (N=65), and 43 birds were seen flushing. No spectacled eiders were observed on dry tundra in either year. Observations were made evenly among the first three distance bins (<150 m), but were lower in the furthest bin (Table 4).

Table 4. Spectacled eider observations in 2017 and 2018 by distance bin. Distance was not recorded for one observation.

| Year | 1 (0-50m) | 2 (50-100m) | 3 (100-150m) | 4 (150-200m) |
| --- | --- | --- | --- | --- |
| 2017 | 41 | 42 | 46 | 16 |
| 2018 | 38 | 43 | 30 | 23 |

The data used in this analysis is given in Appendix B.

*Analysis and Results*

Similar to the approach described for the Steller’s eider data, models were first fit with and without a seat effect. Using the most parsimonious model, as selected by DIC, models with year, distance, front surveyor, transect direction, transect direction and sun angle, group size, and behavior (ground, flush, flight) were then constructed. The best model, as determined by DIC and model weights, was used for inference.

There was no support for the inclusion of a seat effect (equal: DIC=611.2; unequal: DIC=612.4). Of models with sighting covariates, the model including distance was DIC selected (model weight > 95%). Specifically, detection was highest in the range of 50-100m, declining more steeply at further distances. Table 5 presents detection estimates.

Table 5. Estimated detection probabilities for spectacled eiders, 2017 and 2018 as a function of distance. 95% confidence intervals are given in parentheses.

| Distance bin (m) | Detection Estimate |
| --- | --- |
| 1 (0-50m) | 80.7% (74.2% - 86.3%) |
| 2 (50-100m) | 82.7% (76.4% - 87.8%) |
| 3 (100-150m) | 74.5% (67.1% - 81.0%) |
| 4 (150-200m) | 59.1% (47.9% - 69.7%) |

**King Eider**

*Data*

Across both years, solitary male king eiders were most commonly seen (N=166), but 17 pairs, two groups of three, and one group of five were also observed. As with spectacled eiders, group size was treated as a binary variable (one individual or more than one individual). 93 groups were observed by both surveyors and 93 were seen by only one. Of those seen by only one surveyor, 39 were seen by the front seat while 54 were observed from the rear seat.

Covariates as described in the spectacled eider section were collected and used in models of detection probability. King eider data used in the following analysis can be found in Appendix C.

*Analysis and Results*

As described in the spectacled eider section, two models assuming equal and unequal seat effects were first fit to the data and, consistent with the spectacled eider data, no effect of seat was supported (equal: DIC=420.4; unequal: DIC=419.1). A model suite including year, distance, front surveyor, transect direction, transect direction and sun angle, group size, and behavior, were then fit to the data and no support was identified for any sighting variable (DIC ≥ 420.4 for each candidate model). Detection, as estimated from the simplest, most parsimonious, model was estimated as 74.9% (95% CI: 70.4% - 79.1%).

**References**

Barker, R. 2008. Theory and application of mark-recapture and related techniques to aerial surveys of wildlife. Wildlife Research 35: 268-274.

Brooks, SP and A Gelman. 1998. General Methods for Monitoring Convergence of Iterative Simulations. Journal of Computational and Graphical Statistics 7(4): 434-455.

Griffin, PC et al. 2013. A Hybrid Double-Observer Sightability Model for Aerial Surveys. Population Ecology 77(8): 1532-1544.

Huggins, RM. 1989. On the statistical analysis of capture experiments. Biometrika 76: 133-140.

Huggins, RM. 1991. Some practical aspects of a conditional likelihood approach to capture experiments. Biometrics 47: 725-732.

Obritschkewitsch, T and RJ Ritchie. 2017. Steller’s eider surveys near Utqiaġvik, Alaska 2016. Report for Bureau of Land Management, Fairbanks, AK, and U.S. Fish and Wildlife Service, Fairbanks, AK, by ABR, Inc., Fairbanks, AK. 23 pp.

Pearse, AT et al. 2008. Estimation and Correction of Visibility Bias in Aerial Surveys of Wintering Ducks. Journal of Wildlife Mangement 72(3): 808-813.

Pollock, KH and WL Kendall. 1987. Visibility bias in aerial surveys: a review of estimation procedures. Journal of Wildlife Management 70: 255-262.

R Core Team. 2018. R: A language and environment of statistical computing. R Foundation for Statistical Computing, Vienna, Austria.

Steinhorst, RK and MD Samuel. 1989. Sightability adjustment methods for aerial surveys of wildlife populations. Biometrics 45: 415-425.

Strobel, BN and MJ Butler. 2015. Monitoring Whooping Crane Abundance Using Aerial Surveys: Influences on Detectability. Wildlife Society Bulletin 38(1): 188-195.

Sturtz, S, U Ligges, and A Gelman. 2005. R2WinBUGS: A Package for Running WinBUGS from R. Journal of Statistical Software 12(3): 1-16.

Wilson, HM et al. *In prep*. Aerial Survey Detection Rates for Spectacled Eiders on the Arctic Coastal Plain, Alaska.

**Appendix A1. Decoy Deployments, 2017 and 2018.** Easting and Northing values are in UTMs. TransectType: Decoy = transect is not part of the standard survey area; Standard = transect is part of the standard survey area. TransectNo = transect id. NorthSouth: N= decoy placed on the north side of the transect centerline; S= decoy placed on the south side of the transect centerline. DistanceCat = distance category (in meters). HabitatType = 8 types in 2017, 4 types in 2018. Location = tundra, shoreline, off-shore. Bolded decoys are those that were observed by the surveyors during the aerial survey. *Three decoys were not available to the surveyors in 2018 and were dropped from all analyses.

| Year | DecoyID | Easting | Northing | TransectType | TransectNo | NorthSouth | DistanceCat | HabitatType | Location |
| --- | --- | --- | --- | --- | --- | --- | --- | --- | --- |
| 2017 | D01 | 583280.96 | 7908556.96 | Decoy | 1 | S | 130-190 | dry tundra | tundra |
| **2017** | **D02** | **585328.6** | **7907168.87** | **Decoy** | **3** | **S** | **20-80** | **deep-arcto** | **shoreline** |
| **2017** | **D03** | **585807.74** | **7908935.08** | **Decoy** | **1** | **N** | **80-130** | **shallow-arcto** | **off-shore** |
| 2017 | D04 | 589979.74 | 7908884.69 | Decoy | 1 | S | 80-130 | shallow-carex | shoreline |
| **2017** | **D05** | **591493.06** | **7909183.32** | **Decoy** | **1** | **N** | **80-130** | **shallow-carex** | **off-shore** |
| 2017 | D06 | 580775.85 | 7907687.96 | Decoy | 2 | S | 130-190 | deep-arcto | shoreline |
| 2017 | D07 | 588193.94 | 7907978.99 | Decoy | 2 | S | 130-190 | shallow-arcto | shoreline |
| 2017 | D08 | 583045.49 | 7907740.22 | Decoy | 2 | S | 130-190 | shallow-carex | shoreline |
| **2017** | **D09** | **597465.33** | **7908699.94** | **Decoy** | **2** | **N** | **130-190** | **shallow-carex** | **off-shore** |
| **2017** | **D10** | **587330.83** | **7908063.19** | **Decoy** | **2** | **S** | **20-80** | **flooded tundra** | **shoreline** |
| 2017 | D11 | 592117.13 | 7907669.82 | Decoy | 3 | N | 130-190 | shallow-carex | off-shore |
| 2017 | D12 | 597645.5 | 7907578.94 | Decoy | 3 | S | 130-190 | shallow-carex | shoreline |
| 2017 | D13 | 588262.57 | 7907198.67 | Decoy | 3 | S | 130-190 | shallow-carex | shoreline |
| 2017 | D14 | 580980.52 | 7906980.62 | Decoy | 3 | S | 20-80 | dry tundra | tundra |
| 2017 | D15 | 591762.97 | 7907404.24 | Decoy | 3 | S | 80-130 | shallow-arcto | shoreline |
| 2017 | D16 | 593910.35 | 7907473.11 | Decoy | 3 | S | 80-130 | dry tundra | tundra |
| 2017 | D17 | 589356.97 | 7907507.02 | Decoy | 3 | N | 80-130 | ditch | off-shore |
| 2017 | D18 | 589484.33 | 7906763.92 | Decoy | 4 | N | 130-190 | shallow-arcto | off-shore |
| 2017 | D19 | 578970.57 | 7905958.85 | Decoy | 4 | S | 130-190 | shallow-carex | shoreline |
| **2017** | **D20** | **595294.14** | **7907000.72** | **Decoy** | **4** | **N** | **130-190** | **shallow-arcto** | **shoreline** |
| **2017** | **D21** | **582452.9** | **7906241.03** | **Decoy** | **4** | **S** | **20-80** | **deep-open** | **shoreline** |
| **2017** | **D22** | **585090.64** | **7906456.54** | **Decoy** | **4** | **N** | **20-80** | **shallow-arcto** | **off-shore** |
| **2017** | **D23** | **590736.88** | **7906690.29** | **Decoy** | **4** | **N** | **20-80** | **shallow-carex** | **off-shore** |
| 2017 | D24 | 580630.21 | 7906171.53 | Decoy | 4 | S | 20-80 | shallow-arcto | shoreline |
| 2017 | D25 | 586860.57 | 7906419.07 | Decoy | 4 | S | 20-80 | deep-open | shoreline |
| **2017** | **D26** | **592632.23** | **7906825.27** | **Decoy** | **4** | **N** | **80-130** | **shallow-arcto** | **off-shore** |
| 2017 | D27 | 598543.1 | 7906021.19 | Decoy | 5 | S | 130-190 | deep-open | shoreline |
| 2017 | D28 | 582769.85 | 7905476.19 | Decoy | 5 | S | 20-80 | shallow-arcto | shoreline |
| 2017 | D29 | 581785.43 | 7905427.14 | Decoy | 5 | S | 20-80 | ditch | shoreline |
| **2017** | **D30** | **593047.62** | **7905889.35** | **Decoy** | **5** | **S** | **20-80** | **shallow-arcto** | **shoreline** |
| 2017 | D31 | 595619.02 | 7906031.77 | Decoy | 5 | S | 20-80 | stream | shoreline |
| **2017** | **D32** | **585653.29** | **7905561.39** | **Decoy** | **5** | **S** | **20-80** | **deep open** | **shoreline** |
| 2017 | D33 | 587781.06 | 7905657.25 | Decoy | 5 | S | 20-80 | shallow-carex | shoreline |
| 2017 | D34 | 590844.69 | 7905824.92 | Decoy | 5 | S | 20-80 | stream | shoreline |
| 2017 | D35 | 596542.77 | 7906209.86 | Decoy | 5 | N | 80-130 | shallow-arcto | shoreline |
| **2017** | **D36** | **583611.68** | **7905669.64** | **Decoy** | **5** | **N** | **80-130** | **shallow-arcto** | **tundra** |
| **2017** | **D37** | **587258.83** | **7905792.24** | **Decoy** | **5** | **N** | **80-130** | **deep-arcto** | **off-shore** |
| 2017 | SD38 | 587847.27 | 7906841.66 | Standard | 4 | S | 80-130 | deep-arcto | off-shore |
| **2017** | **SD39** | **595914.87** | **7904811.58** | **Standard** | **7** | **S** | **20-80** | **shallow-arcto** | **shoreline** |
| 2017 | SD40 | 580809.7 | 7908130.75 | Standard | 2 | S | 80-130 | shallow-carex | shoreline |
| **2018** | **D1** | **583561.33** | **7908766.38** | **Standard** | **1** | **N** | **20-50** | **shallow pond** | **shoreline** |
| **2018** | **D2** | **586569.85** | **7908729.32** | **Standard** | **1** | **S** | **100-150** | **shallow pond** | **shoreline** |
| 2018 | D3 | 577516.94 | 7907585.1 | Standard | 2 | S | 50-100 | ground | tundra |
| **2018** | **D4** | **580880.6** | **7907735.68** | **Standard** | **2** | **S** | **50-100** | **shallow pond** | **shoreline** |
| 2018 | D5 | 584937.18 | 7907035.86 | Standard | 3 | S | 150-180 | deep pond | shoreline |
| **2018** | **D6** | **591373.22** | **7907436.23** | **Standard** | **3** | **S** | **20-50** | **shallow pond** | **shoreline** |
| 2018 | D7 | 592925.12 | 7907471.31 | Standard | 3 | S | 50-100 | shallow pond | shoreline |
| **2018** | **D8** | **581257.25** | **7907140.6** | **Standard** | **3** | **N** | **50-100** | **shallow pond** | **shoreline** |
| 2018 | D9 | 587560.11 | 7907477.37 | Standard | 3 | N | 150-180 | shallow pond | shoreline |
| 2018 | D10 | 583967.94 | 7907037.25 | Standard | 3 | S | 100-150 | ground | tundra |
| **2018** | **D11** | **588394.65** | **7907379.01** | **Standard** | **3** | **N** | **20-50** | **deep pond** | **shoreline** |
| **2018** | **D12** | **588583.42** | **7907277.84** | **Standard** | **3** | **S** | **50-100** | **shallow pond** | **shoreline** |
| 2018 | D13 | 595383.76 | 7907478.97 | Standard | 3 | S | 150-180 | deep pond | shoreline |
| 2018 | D14 | 586613.6 | 7907142.27 | Standard | 3 | S | 100-150 | shallow pond | shoreline |
| 2018 | D15 | 585040.45 | 7906531.91 | Standard | 4 | N | 100-150 | shallow pond | shoreline |
| **2018** | **D16** | **588447.12** | **7906505.02** | **Standard** | **4** | **S** | **20-50** | **shallow pond** | **shoreline** |
| 2018 | D17 | 592845.88 | 7906876.62 | Standard | 4 | N | 100-150 | shallow pond | shoreline |
| **2018** | **D18** | **581187.85** | **7905580.74** | **Standard** | **5** | **N** | **100-150** | **deep pond** | **shoreline** |
| **2018** | **D19** | **593114.67** | **7906770.59** | **Standard** | **4** | **N** | **20-50** | **deep pond** | **shoreline** |
| 2018 | D20 | 588067.59 | 7906409.45 | Standard | 4 | S | 100-150 | shallow pond | shoreline |
| **2018** | **D21** | **593746.08** | **7906739.32** | **Standard** | **4** | **S** | **20-50** | **shallow pond** | **shoreline** |
| 2018 | D22 | 591140.99 | 7905704.79 | Standard | 5 | S | 150-180 | shallow pond | shoreline |
| **2018** | **D23** | **588629.46** | **7905883.13** | **Standard** | **5** | **N** | **100-150** | **shallow pond** | **shoreline** |
| **2018** | **D24** | **591584** | **7905968.64** | **Standard** | **5** | **N** | **50-100** | **ground** | **tundra** |
| **2018** | **D25** | **585723.2** | **7905594.97** | **Standard** | **5** | **S** | **20-50** | **shallow pond** | **shoreline** |
| 2018 | D26 | 590466.21 | 7905997.98 | Standard | 5 | N | 150-180 | shallow pond | shoreline |
| 2018 | D27 | 596663.15 | 7906072.35 | Standard | 5 | S | 20-50 | ground | tundra |
| **2018** | **D28** | **594759.48** | **7906001.17** | **Standard** | **5** | **S** | **20-50** | **deep pond** | **shoreline** |
| 2018 | D29 | 596429.5 | 7906230.61 | Standard | 5 | N | 100-150 | shallow pond | shoreline |
| 2018 | D30 | 581283.87 | 7905604.95 | Standard | 5 | N | 150-180 | shallow pond | shoreline |
| 2018 | D31 | 591458.3 | 7905923.74 | Standard | 5 | N | 20-50 | shallow pond | shoreline |
| 2018 | D32 | 590726.57 | 7905690.84 | Standard | 5 | S | 150-180 | ground | tundra |
| 2018 | D33 | 595240.13 | 7906014.21 | Standard | 5 | S | 20-50 | shallow pond | shoreline |
| 2018 | D34* | 595013.91 | 7905153.38 | Standard | 6 | S | 50-100 | stream | shoreline |
| 2018 | D35* | 595917.92 | 7905084.57 | Standard | 6 | S | 150-180 | shallow pond | shoreline |
| **2018** | **D36** | **588731.41** | **7908225.55** | **Standard** | **2** | **N** | **100-150** | **deep pond** | **shoreline** |
| 2018 | D37* | 594600.62 | 7905063.69 | Standard | 6 | S | 100-150 | shallow pond | shoreline |
| 2018 | D38 | 596959.55 | 7905331.52 | Standard | 6 | N | 20-50 | shallow pond | shoreline |
| 2018 | D39 | 589818.38 | 7907539.77 | Standard | 2 | N | 100-150 | deep pond | shoreline |
| 2018 | D40 | 595854.14 | 7905302.22 | Standard | 6 | N | 20-50 | shallow pond | shoreline |

**Appendix A2. Double-observer sightability (DOS) aerial survey Steller’s eider data, 2017 and 2018.** ObsType = Decoy or STEI. Behavior = Ground or Air. Distance = 3 bins in 2017 (1 = 20-80m, 2 = 80-130m, 3 = 130-190m); = 4 bins in 2018 (1 = 0-50m, 2 = 50-100m, 3 = 100-150m, 4 = 150- 200m). Front = observation from the front seat (1 = yes, 0 = no). Rear = observation from the rear seat (1 = yes, 0 = no). Decoy = observation is conditional (0) or unconditional (1) on at least one surveyor seeing the decoy/bird*.*  *Note that data in the decoy deployment table (Appendix A1) and data for decoys in this table can differ. This can be due to changes in habitat such as water receding or distance discrepancies because of differences in planned and actual flight lines.* FrontObs, RearObs = identity of front and rear surveyors. TransDir = transect-level variable, direction of the transect. SunDegree = transect-level variable, with due North as 0, the predominate direction from which the sun was hitting the airplane during the transect.

| Year | ObsType | DecoyID | Behavior | Habitat | Distance | Front | Rear | Decoy | FrontObs | RearObs | TransDir | SunDegree |
| --- | --- | --- | --- | --- | --- | --- | --- | --- | --- | --- | --- | --- |
| 2017 | DECOY | D01 | Ground | Ground | 3 | 0 | 0 | 1 | JES | TO | East | 135 |
| 2017 | DECOY | D02 | Ground | DeepPond | 3 | 0 | 1 | 1 | JES | TO | East | 135 |
| 2017 | DECOY | D03 | Ground | ShallowPond | 2 | 1 | 1 | 1 | TO | JES | West | 270 |
| 2017 | DECOY | D04 | Ground | ShallowPond | 2 | 0 | 0 | 1 | TO | JES | West | 270 |
| 2017 | DECOY | D05 | Ground | ShallowPond | 2 | 1 | 0 | 1 | TO | JES | West | 270 |
| 2017 | DECOY | D06 | Ground | DeepPond | 3 | 0 | 0 | 1 | JES | TO | West | 145 |
| 2017 | DECOY | D07 | Ground | ShallowPond | 3 | 0 | 0 | 1 | TO | JES | East | 285 |
| 2017 | DECOY | D08 | Ground | ShallowPond | 3 | 0 | 0 | 1 | JES | TO | West | 145 |
| 2017 | DECOY | D09 | Ground | ShallowPond | 3 | 1 | 0 | 1 | TO | JES | East | 285 |
| 2017 | DECOY | D10 | Ground | ShallowPond | 1 | 0 | 1 | 1 | TO | JES | East | 285 |
| 2017 | DECOY | D11 | Ground | ShallowPond | 3 | 0 | 0 | 1 | JES | TO | East | 135 |
| 2017 | DECOY | D12 | Ground | ShallowPond | 3 | 0 | 0 | 1 | TO | JES | West | 285 |
| 2017 | DECOY | D13 | Ground | ShallowPond | 3 | 0 | 0 | 1 | TO | JES | West | 285 |
| 2017 | DECOY | D14 | Ground | Ground | 1 | 0 | 0 | 1 | JES | TO | East | 135 |
| 2017 | DECOY | D15 | Ground | ShallowPond | 2 | 0 | 0 | 1 | JES | TO | East | 135 |
| 2017 | DECOY | D16 | Ground | Ground | 2 | 0 | 0 | 1 | TO | JES | West | 285 |
| 2017 | DECOY | D17 | Ground | Stream | 2 | 0 | 0 | 1 | JES | TO | East | 135 |
| 2017 | DECOY | D18 | Ground | ShallowPond | 3 | 0 | 0 | 1 | TO | JES | East | 285 |
| 2017 | DECOY | D19 | Ground | ShallowPond | 3 | 0 | 0 | 1 | TO | JES | East | 285 |
| 2017 | DECOY | D20 | Ground | ShallowPond | 3 | 0 | 1 | 1 | JES | TO | West | 120 |
| 2017 | DECOY | D21 | Ground | DeepPond | 1 | 1 | 1 | 1 | TO | JES | East | 285 |
| 2017 | DECOY | D22 | Ground | ShallowPond | 1 | 1 | 1 | 1 | JES | TO | West | 120 |
| 2017 | DECOY | D23 | Ground | ShallowPond | 1 | 1 | 1 | 1 | JES | TO | West | 120 |
| 2017 | DECOY | D24 | Ground | ShallowPond | 1 | 0 | 0 | 1 | TO | JES | East | 285 |
| 2017 | DECOY | D25 | Ground | DeepPond | 1 | 0 | 0 | 1 | TO | JES | East | 285 |
| 2017 | DECOY | D26 | Ground | ShallowPond | 2 | 1 | 1 | 1 | JES | TO | West | 120 |
| 2017 | DECOY | D27 | Ground | DeepPond | 3 | 0 | 0 | 1 | JES | TO | East | 150 |
| 2017 | DECOY | D28 | Ground | ShallowPond | 1 | 0 | 0 | 1 | JES | TO | East | 150 |
| 2017 | DECOY | D29 | Ground | Stream | 1 | 0 | 0 | 1 | JES | TO | East | 150 |
| 2017 | DECOY | D30 | Ground | ShallowPond | 1 | 1 | 0 | 1 | JES | TO | East | 150 |
| 2017 | DECOY | D31 | Ground | Stream | 1 | 0 | 0 | 1 | TO | JES | West | 285 |
| 2017 | DECOY | D32 | Ground | ShallowPond | 1 | 1 | 0 | 1 | JES | TO | East | 150 |
| 2017 | DECOY | D33 | Ground | ShallowPond | 1 | 0 | 0 | 1 | TO | JES | West | 285 |
| 2017 | DECOY | D34 | Ground | Stream | 1 | 0 | 0 | 1 | TO | JES | West | 285 |
| 2017 | DECOY | D35 | Ground | ShallowPond | 2 | 0 | 0 | 1 | JES | TO | East | 150 |
| 2017 | DECOY | D36 | Ground | ShallowPond | 2 | 0 | 1 | 1 | JES | TO | East | 150 |
| 2017 | DECOY | D37 | Ground | DeepPond | 2 | 0 | 1 | 1 | TO | JES | West | 285 |
| 2017 | DECOY | D38 | Ground | DeepPond | 2 | 0 | 0 | 1 | TO | JES | West | 270 |
| 2017 | DECOY | D39 | Ground | ShallowPond | 1 | 0 | 1 | 1 | TO | JES | East | 255 |
| 2017 | DECOY | D40 | Ground | ShallowPond | 2 | 0 | 0 | 1 | TO | JES | West | 270 |
| 2017 | STEI | NA | Ground | ShallowPond | 1 | 1 | 1 | 0 | JES | TO | East | 135 |
| 2017 | STEI | NA | Ground | DeepPond | 1 | 1 | 0 | 0 | TO | JES | East | 255 |
| 2017 | STEI | NA | Ground | ShallowPond | 1 | 1 | 0 | 0 | JES | TO | East | 255 |
| 2017 | STEI | NA | Ground | ShallowPond | 1 | 1 | 0 | 0 | JES | TO | West | 120 |
| 2017 | STEI | NA | Ground | ShallowPond | 1 | 1 | 0 | 0 | JES | TO | West | 145 |
| 2017 | STEI | NA | Ground | Stream | 1 | 0 | 1 | 0 | JES | TO | West | 145 |
| 2017 | STEI | NA | Ground | Stream | 1 | 0 | 1 | 0 | TO | JES | West | 270 |
| 2017 | STEI | NA | Ground | ShallowPond | 1 | 0 | 1 | 0 | TO | JES | West | 285 |
| 2017 | STEI | NA | Air | Air | 2 | 1 | 1 | 0 | JES | TO | East | 135 |
| 2017 | STEI | NA | Air | Air | 2 | 0 | 1 | 0 | TO | JES | East | 255 |
| 2017 | STEI | NA | Ground | ShallowPond | 2 | 1 | 0 | 0 | TO | JES | West | 270 |
| 2017 | STEI | NA | Air | Air | 2 | 1 | 1 | 0 | TO | JES | West | 285 |
| 2017 | STEI | NA | Air | Air | 3 | 1 | 1 | 0 | JES | TO | East | 240 |
| 2017 | STEI | NA | Air | Air | 3 | 1 | 0 | 0 | JES | TO | West | 145 |
| 2018 | DECOY | D01 | Ground | ShallowPond | 1 | 1 | 1 | 1 | TO | JES | West | 105 |
| 2018 | DECOY | D02 | Ground | ShallowPond | 3 | 1 | 1 | 1 | JES | TO | East | 150 |
| 2018 | DECOY | D03 | Ground | Ground | 2 | 0 | 0 | 1 | TO | JES | East | 105 |
| 2018 | DECOY | D04 | Ground | ShallowPond | 2 | 1 | 1 | 1 | TO | JES | East | 105 |
| 2018 | DECOY | D05 | Ground | DeepPond | 4 | 0 | 0 | 1 | JES | TO | East | 150 |
| 2018 | DECOY | D06 | Ground | ShallowPond | 1 | 1 | 1 | 1 | JES | TO | East | 150 |
| 2018 | DECOY | D07 | Ground | ShallowPond | 2 | 0 | 0 | 1 | JES | TO | East | 150 |
| 2018 | DECOY | D08 | Ground | ShallowPond | 2 | 1 | 0 | 1 | TO | JES | West | 105 |
| 2018 | DECOY | D09 | Ground | ShallowPond | 4 | 0 | 0 | 1 | TO | JES | West | 105 |
| 2018 | DECOY | D10 | Ground | Ground | 3 | 0 | 0 | 1 | JES | TO | East | 150 |
| 2018 | DECOY | D11 | Ground | DeepPond | 1 | 1 | 0 | 1 | TO | JES | West | 105 |
| 2018 | DECOY | D12 | Ground | ShallowPond | 2 | 1 | 1 | 1 | JES | TO | East | 150 |
| 2018 | DECOY | D13 | Ground | DeepPond | 4 | 0 | 0 | 1 | JES | TO | East | 150 |
| 2018 | DECOY | D14 | Ground | ShallowPond | 3 | 0 | 0 | 1 | JES | TO | East | 150 |
| 2018 | DECOY | D15 | Ground | ShallowPond | 3 | 0 | 0 | 1 | JES | TO | West | 135 |
| 2018 | DECOY | D16 | Ground | ShallowPond | 1 | 1 | 1 | 1 | TO | JES | East | 105 |
| 2018 | DECOY | D17 | Ground | ShallowPond | 3 | 0 | 0 | 1 | JES | TO | West | 135 |
| 2018 | DECOY | D18 | Ground | DeepPond | 3 | 0 | 0 | 1 | TO | JES | West | 120 |
| 2018 | DECOY | D19 | Ground | DeepPond | 1 | 1 | 0 | 1 | JES | TO | West | 135 |
| 2018 | DECOY | D20 | Ground | ShallowPond | 3 | 0 | 0 | 1 | TO | JES | East | 105 |
| 2018 | DECOY | D21 | Ground | ShallowPond | 1 | 1 | 1 | 1 | TO | JES | East | 105 |
| 2018 | DECOY | D22 | Ground | ShallowPond | 4 | 0 | 0 | 1 | JES | TO | East | 135 |
| 2018 | DECOY | D23 | Ground | ShallowPond | 3 | 1 | 1 | 1 | TO | JES | West | 120 |
| 2018 | DECOY | D24 | Ground | Ground | 2 | 1 | 1 | 1 | TO | JES | West | 120 |
| 2018 | DECOY | D25 | Ground | ShallowPond | 1 | 1 | 1 | 1 | JES | TO | East | 135 |
| 2018 | DECOY | D26 | Ground | ShallowPond | 4 | 0 | 0 | 1 | TO | JES | West | 120 |
| 2018 | DECOY | D27 | Ground | Ground | 1 | 0 | 0 | 1 | JES | TO | East | 135 |
| 2018 | DECOY | D28 | Ground | DeepPond | 1 | 1 | 0 | 1 | JES | TO | East | 135 |
| 2018 | DECOY | D29 | Ground | ShallowPond | 3 | 0 | 0 | 1 | TO | JES | West | 120 |
| 2018 | DECOY | D30 | Ground | ShallowPond | 4 | 0 | 0 | 1 | TO | JES | West | 120 |
| 2018 | DECOY | D31 | Ground | ShallowPond | 1 | 0 | 0 | 1 | TO | JES | West | 120 |
| 2018 | DECOY | D32 | Ground | Ground | 4 | 0 | 0 | 1 | JES | TO | East | 135 |
| 2018 | DECOY | D33 | Ground | ShallowPond | 1 | 0 | 0 | 1 | JES | TO | East | 135 |
| 2018 | DECOY | D36 | Ground | DeepPond | 3 | 0 | 0 | 1 | JES | TO | West | 150 |
| 2018 | DECOY | D38 | Ground | ShallowPond | 1 | 0 | 0 | 1 | JES | TO | West | 165 |
| 2018 | DECOY | D39 | Ground | DeepPond | 3 | 0 | 0 | 1 | JES | TO | West | 150 |
| 2018 | DECOY | D40 | Ground | ShallowPond | 1 | 0 | 0 | 1 | JES | TO | West | 165 |
| 2018 | STEI | NA | Air | Air | 1 | 1 | 1 | 0 | JES | TO | East | 165 |
| 2018 | STEI | NA | Ground | ShallowPond | 1 | 1 | 1 | 0 | JES | TO | West | 150 |
| 2018 | STEI | NA | Ground | ShallowPond | 1 | 1 | 1 | 0 | JES | TO | West | 150 |
| 2018 | STEI | NA | Ground | Tundra | 2 | 1 | 0 | 0 | TO | JES | West | 105 |
| 2018 | STEI | NA | Ground | ShallowPond | 2 | 0 | 1 | 0 | TO | JES | West | 105 |
| 2018 | STEI | NA | Air | Air | 3 | 1 | 1 | 0 | JES | TO | East | 150 |
| 2018 | STEI | NA | Ground | ShallowPond | 3 | 1 | 0 | 0 | TO | JES | West | 105 |
| 2018 | STEI | NA | Ground | ShallowPond | 3 | 1 | 0 | 0 | TO | JES | West | 105 |
| 2018 | STEI | NA | Ground | ShallowPond | 3 | 1 | 0 | 0 | TO | JES | West | 105 |
| 2018 | STEI | NA | Air | Air | 3 | 0 | 1 | 0 | JES | TO | West | 225 |
| 2018 | STEI | NA | Ground | ShallowPond | 4 | 1 | 1 | 0 | TO | JES | East | 105 |
| 2018 | STEI | NA | Ground | Stream | 4 | 1 | 1 | 0 | TO | JES | West | 105 |

**Appendix B. Double-observer (DO) aerial survey spectacled eider data, 2017 and 2018.** Males = number of males observed in the group. Behavior = Flying, Flush, or Ground (neither flying nor flushing). Habitat = 5 types (air, shallow pond, deep pond, ground, or stream). Distance = 4 bins; 1 = 0-50m, 2 = 51-100m, 3 = 101-150m, 4 = 151-200m. Front = observation from front seat (1 = yes, 0 = no). Rear = observation from rear seat (1 = yes, 0 = no). FrontObs, RearObs = identity of front and rear surveyors. TransDir = transect-level variable, direction of the transect. SunDegree = transect-level variable, with due North as 0, the predominate direction from which the sun was hitting the airplane during the transect.

| Year | Males | Behavior | Habitat | Distance | Front | Rear | FrontObs | RearObs | TransDir | SunDegree |
| --- | --- | --- | --- | --- | --- | --- | --- | --- | --- | --- |
| 2017 | 1 | Flush | ShallowPond | 2 | 0 | 1 | JES | TO | East | 135 |
| 2017 | 1 | Ground | ShallowPond | 2 | 0 | 1 | JES | TO | East | 150 |
| 2017 | 1 | Flying | Air | 1 | 0 | 1 | TO | JES | East | 210 |
| 2017 | 1 | Flying | ShallowPond | 1 | 0 | 1 | TO | JES | East | 210 |
| 2017 | 1 | Flying | ShallowPond | 1 | 0 | 1 | TO | JES | East | 210 |
| 2017 | 1 | Ground | ShallowPond | 3 | 0 | 1 | JES | TO | East | 210 |
| 2017 | 1 | Flying | Air | 4 | 0 | 1 | JES | TO | East | 210 |
| 2017 | 1 | Flying | Air | 2 | 0 | 1 | JES | TO | East | 225 |
| 2017 | 1 | Flying | Air | 3 | 0 | 1 | JES | TO | East | 225 |
| 2017 | 1 | Flush | ShallowPond | 3 | 0 | 1 | TO | JES | East | 225 |
| 2017 | 1 | Ground | DeepPond | 3 | 0 | 1 | TO | JES | East | 240 |
| 2017 | 1 | Flying | Air | 4 | 0 | 1 | JES | TO | East | 240 |
| 2017 | 1 | Flush | ShallowPond | 4 | 0 | 1 | JES | TO | East | 240 |
| 2017 | 1 | Ground | ShallowPond | 1 | 0 | 1 | TO | JES | East | 255 |
| 2017 | 1 | Ground | ShallowPond | 1 | 0 | 1 | JES | TO | West | 270 |
| 2017 | 1 | Ground | ShallowPond | 4 | 0 | 1 | JES | TO | West | 255 |
| 2017 | 1 | Flush | ShallowPond | 3 | 0 | 1 | JES | TO | West | 240 |
| 2017 | 1 | Flush | ShallowPond | 1 | 0 | 1 | TO | JES | West | 225 |
| 2017 | 1 | Ground | ShallowPond | 1 | 0 | 1 | JES | TO | West | 210 |
| 2017 | 1 | Flush | ShallowPond | 2 | 0 | 1 | JES | TO | West | 210 |
| 2017 | 1 | Ground | ShallowPond | 3 | 0 | 1 | JES | TO | West | 210 |
| 2017 | 1 | Ground | ShallowPond | 2 | 0 | 1 | JES | TO | West | 195 |
| 2017 | 1 | Flying | Air | 1 | 0 | 1 | TO | JES | West | 165 |
| 2017 | 1 | Flying | Air | 3 | 0 | 1 | JES | TO | West | 165 |
| 2017 | 1 | Ground | ShallowPond | 2 | 0 | 1 | JES | TO | West | 150 |
| 2017 | 1 | Flying | Air | 4 | 0 | 1 | JES | TO | West | 135 |
| 2017 | 2 | Flying | Air | 3 | 0 | 1 | JES | TO | East | 150 |
| 2017 | 2 | Ground | ShallowPond | 2 | 0 | 1 | TO | JES | East | 165 |
| 2017 | 2 | Ground | ShallowPond | 4 | 0 | 1 | JES | TO | East | 240 |
| 2017 | 3 | Ground | ShallowPond | 2 | 0 | 1 | TO | JES | East | 165 |
| 2017 | 3 | Ground | ShallowPond | 3 | 0 | 1 | TO | JES | East | 210 |
| 2017 | 1 | Ground | ShallowPond | 3 | 1 | 0 | JES | TO | East | 150 |
| 2017 | 1 | Flush | ShallowPond | 4 | 1 | 0 | TO | JES | East | 150 |
| 2017 | 1 | Ground | ShallowPond | 1 | 1 | 0 | TO | JES | East | 165 |
| 2017 | 1 | Ground | ShallowPond | 4 | 1 | 0 | TO | JES | East | 165 |
| 2017 | 1 | Flush | ShallowPond | 4 | 1 | 0 | TO | JES | East | 165 |
| 2017 | 1 | Flying | Air | 1 | 1 | 0 | TO | JES | East | 210 |
| 2017 | 1 | Ground | ShallowPond | 2 | 1 | 0 | TO | JES | East | 210 |
| 2017 | 1 | Flying | ShallowPond | 3 | 1 | 0 | JES | TO | East | 210 |
| 2017 | 1 | Ground | ShallowPond | 1 | 1 | 0 | JES | TO | East | 225 |
| 2017 | 1 | Flying | ShallowPond | 3 | 1 | 0 | JES | TO | East | 240 |
| 2017 | 1 | Ground | ShallowPond | 3 | 1 | 0 | JES | TO | East | 240 |
| 2017 | 1 | Flying | ShallowPond | 3 | 1 | 0 | JES | TO | East | 255 |
| 2017 | 1 | Ground | ShallowPond | 3 | 1 | 0 | JES | TO | East | 255 |
| 2017 | 1 | Ground | ShallowPond | 3 | 1 | 0 | JES | TO | West | 270 |
| 2017 | 1 | Flying | ShallowPond | 4 | 1 | 0 | JES | TO | West | 270 |
| 2017 | 1 | Flying | Air | 1 | 1 | 0 | TO | JES | West | 255 |
| 2017 | 1 | Ground | ShallowPond | 1 | 1 | 0 | TO | JES | West | 255 |
| 2017 | 1 | Ground | ShallowPond | 3 | 1 | 0 | JES | TO | West | 255 |
| 2017 | 1 | Flying | Air | 4 | 1 | 0 | JES | TO | West | 255 |
| 2017 | 1 | Flying | Air | 2 | 1 | 0 | TO | JES | West | 240 |
| 2017 | 1 | Ground | ShallowPond | 2 | 1 | 0 | JES | TO | West | 240 |
| 2017 | 1 | Ground | ShallowPond | 3 | 1 | 0 | JES | TO | West | 240 |
| 2017 | 1 | Ground | ShallowPond | 1 | 1 | 0 | TO | JES | West | 225 |
| 2017 | 1 | Ground | ShallowPond | 3 | 1 | 0 | JES | TO | West | 225 |
| 2017 | 1 | Ground | ShallowPond | 1 | 1 | 0 | JES | TO | West | 210 |
| 2017 | 1 | Flying | Air | 3 | 1 | 0 | JES | TO | West | 210 |
| 2017 | 1 | Ground | ShallowPond | 3 | 1 | 0 | JES | TO | West | 210 |
| 2017 | 1 | Flush | ShallowPond | 1 | 1 | 0 | JES | TO | West | 195 |
| 2017 | 1 | Flying | Air | 3 | 1 | 0 | JES | TO | West | 180 |
| 2017 | 1 | Flying | Air | 3 | 1 | 0 | JES | TO | West | 180 |
| 2017 | 1 | Flying | Air | 4 | 1 | 0 | JES | TO | West | 180 |
| 2017 | 1 | Flying | Air | 1 | 1 | 0 | JES | TO | West | 150 |
| 2017 | 1 | Ground | ShallowPond | 2 | 1 | 0 | JES | TO | West | 150 |
| 2017 | 1 | Ground | ShallowPond | 2 | 1 | 0 | TO | JES | West | 150 |
| 2017 | 2 | Flush | ShallowPond | 2 | 1 | 0 | TO | JES | East | 165 |
| 2017 | 2 | Flying | Air | 2 | 1 | 0 | TO | JES | East | 210 |
| 2017 | 2 | Flush | Stream | 1 | 1 | 0 | JES | TO | West | 180 |
| 2017 | 1 | Ground | ShallowPond | 1 | 1 | 1 | JES | TO | East | 135 |
| 2017 | 1 | Ground | ShallowPond | 2 | 1 | 1 | JES | TO | East | 135 |
| 2017 | 1 | Flush | ShallowPond | 3 | 1 | 1 | JES | TO | East | 135 |
| 2017 | 1 | Flying | Air | 1 | 1 | 1 | JES | TO | East | 150 |
| 2017 | 1 | Flush | ShallowPond | 1 | 1 | 1 | JES | TO | East | 150 |
| 2017 | 1 | Flying | Air | 3 | 1 | 1 | JES | TO | East | 150 |
| 2017 | 1 | Flying | Air | 3 | 1 | 1 | JES | TO | East | 150 |
| 2017 | 1 | Ground | ShallowPond | 3 | 1 | 1 | TO | JES | East | 150 |
| 2017 | 1 | Flush | ShallowPond | 1 | 1 | 1 | JES | TO | East | 210 |
| 2017 | 1 | Flush | ShallowPond | 1 | 1 | 1 | JES | TO | East | 210 |
| 2017 | 1 | Ground | ShallowPond | 1 | 1 | 1 | TO | JES | East | 210 |
| 2017 | 1 | Ground | ShallowPond | 2 | 1 | 1 | TO | JES | East | 210 |
| 2017 | 1 | Flying | Air | 3 | 1 | 1 | TO | JES | East | 210 |
| 2017 | 1 | Flush | ShallowPond | 3 | 1 | 1 | JES | TO | East | 210 |
| 2017 | 1 | Flush | ShallowPond | 3 | 1 | 1 | TO | JES | East | 210 |
| 2017 | 1 | Ground | ShallowPond | 3 | 1 | 1 | TO | JES | East | 210 |
| 2017 | 1 | Ground | ShallowPond | 2 | 1 | 1 | TO | JES | East | 240 |
| 2017 | 1 | Flush | ShallowPond | 2 | 1 | 1 | JES | TO | East | 240 |
| 2017 | 1 | Flush | ShallowPond | 2 | 1 | 1 | JES | TO | East | 240 |
| 2017 | 1 | Ground | ShallowPond | 2 | 1 | 1 | JES | TO | East | 240 |
| 2017 | 1 | Ground | ShallowPond | 2 | 1 | 1 | JES | TO | East | 240 |
| 2017 | 1 | Flying | Air | 3 | 1 | 1 | JES | TO | East | 240 |
| 2017 | 1 | Flying | DeepPond | 1 | 1 | 1 | TO | JES | East | 255 |
| 2017 | 1 | Flush | ShallowPond | 1 | 1 | 1 | JES | TO | East | 255 |
| 2017 | 1 | Flush | ShallowPond | 2 | 1 | 1 | JES | TO | East | 255 |
| 2017 | 1 | Ground | ShallowPond | 2 | 1 | 1 | TO | JES | East | 255 |
| 2017 | 1 | Ground | ShallowPond | 3 | 1 | 1 | JES | TO | East | 255 |
| 2017 | 1 | Ground | ShallowPond | 1 | 1 | 1 | TO | JES | East | 270 |
| 2017 | 1 | Flying | Air | 3 | 1 | 1 | JES | TO | East | 270 |
| 2017 | 1 | Flush | ShallowPond | 3 | 1 | 1 | JES | TO | East | 270 |
| 2017 | 1 | Ground | ShallowPond | 3 | 1 | 1 | JES | TO | East | 270 |
| 2017 | 1 | Ground | DeepPond | 2 | 1 | 1 | TO | JES | East | 285 |
| 2017 | 1 | Ground | ShallowPond | 1 | 1 | 1 | TO | JES | West | 285 |
| 2017 | 1 | Flying | Air | 2 | 1 | 1 | TO | JES | West | 285 |
| 2017 | 1 | Ground | ShallowPond | 2 | 1 | 1 | TO | JES | West | 285 |
| 2017 | 1 | Ground | ShallowPond | 3 | 1 | 1 | TO | JES | West | 285 |
| 2017 | 1 | Ground | ShallowPond | 1 | 1 | 1 | TO | JES | West | 270 |
| 2017 | 1 | Ground | ShallowPond | 2 | 1 | 1 | JES | TO | West | 270 |
| 2017 | 1 | Flying | Air | 1 | 1 | 1 | TO | JES | West | 255 |
| 2017 | 1 | Flush | ShallowPond | 2 | 1 | 1 | JES | TO | West | 255 |
| 2017 | 1 | Flush | ShallowPond | 1 | 1 | 1 | TO | JES | West | 240 |
| 2017 | 1 | Ground | ShallowPond | 2 | 1 | 1 | JES | TO | West | 240 |
| 2017 | 1 | Ground | ShallowPond | 1 | 1 | 1 | JES | TO | West | 225 |
| 2017 | 1 | Flush | ShallowPond | 2 | 1 | 1 | TO | JES | West | 225 |
| 2017 | 1 | Flying | Air | 3 | 1 | 1 | TO | JES | West | 225 |
| 2017 | 1 | Flush | ShallowPond | 4 | 1 | 1 | TO | JES | West | 225 |
| 2017 | 1 | Flush | ShallowPond | 1 | 1 | 1 | JES | TO | West | 210 |
| 2017 | 1 | Ground | ShallowPond | 1 | 1 | 1 | JES | TO | West | 210 |
| 2017 | 1 | Ground | ShallowPond | 2 | 1 | 1 | JES | TO | West | 210 |
| 2017 | 1 | Flush | ShallowPond | 3 | 1 | 1 | JES | TO | West | 210 |
| 2017 | 1 | Flush | ShallowPond | 1 | 1 | 1 | TO | JES | West | 195 |
| 2017 | 1 | Flying | Air | 2 | 1 | 1 | JES | TO | West | 195 |
| 2017 | 1 | Flush | ShallowPond | 2 | 1 | 1 | JES | TO | West | 195 |
| 2017 | 1 | Ground | ShallowPond | 3 | 1 | 1 | TO | JES | West | 195 |
| 2017 | 1 | Flush | DeepPond | 1 | 1 | 1 | JES | TO | West | 180 |
| 2017 | 1 | Ground | ShallowPond | 2 | 1 | 1 | TO | JES | West | 180 |
| 2017 | 1 | Ground | ShallowPond | 4 | 1 | 1 | JES | TO | West | 180 |
| 2017 | 1 | Flying | Air | 2 | 1 | 1 | TO | JES | West | 165 |
| 2017 | 1 | Flush | ShallowPond | 2 | 1 | 1 | JES | TO | West | 165 |
| 2017 | 1 | Flush | ShallowPond | 2 | 1 | 1 | TO | JES | West | 165 |
| 2017 | 1 | Flush | ShallowPond | 3 | 1 | 1 | JES | TO | West | 165 |
| 2017 | 1 | Ground | ShallowPond | 4 | 1 | 1 | TO | JES | West | 165 |
| 2017 | 1 | Flying | Air | 1 | 1 | 1 | JES | TO | West | 150 |
| 2017 | 1 | Flush | ShallowPond | 3 | 1 | 1 | JES | TO | West | 150 |
| 2017 | 1 | Ground | ShallowPond | 4 | 1 | 1 | TO | JES | West | 150 |
| 2017 | 2 | Ground | ShallowPond | 1 | 1 | 1 | JES | TO | East | 150 |
| 2017 | 2 | Ground | ShallowPond | 2 | 1 | 1 | JES | TO | East | 150 |
| 2017 | 2 | Flush | ShallowPond | 3 | 1 | 1 | JES | TO | East | 195 |
| 2017 | 2 | Ground | ShallowPond | 1 | 1 | 1 | JES | TO | East | 240 |
| 2017 | 2 | Ground | ShallowPond | 2 | 1 | 1 | TO | JES | East | 255 |
| 2017 | 2 | Flush | ShallowPond | 1 | 1 | 1 | JES | TO | West | 270 |
| 2017 | 2 | Flying | Air | 1 | 1 | 1 | JES | TO | West | 150 |
| 2017 | 2 | Ground | ShallowPond | 2 | 1 | 1 | JES | TO | West | 150 |
| 2017 | 2 | Flush | ShallowPond | 3 | 1 | 1 | JES | TO | West | 150 |
| 2017 | 3 | Flush | ShallowPond | 2 | 1 | 1 | JES | TO | East | 195 |
| 2017 | 3 | Flying | Air | 3 | 1 | 1 | JES | TO | East | 225 |
| 2017 | 3 | Flush | ShallowPond | 3 | 1 | 1 | TO | JES | West | 165 |
| 2018 | 1 | Flying | Air | 4 | 0 | 1 | JES | TO | East | 135 |
| 2018 | 1 | Ground | ShallowPond | 3 | 0 | 1 | TO | JES | East | 150 |
| 2018 | 1 | Flush | DeepPond | 1 | 0 | 1 | JES | TO | East | 150 |
| 2018 | 1 | Ground | ShallowPond | 1 | 0 | 1 | TO | JES | East | 180 |
| 2018 | 1 | Flush | ShallowPond | 2 | 0 | 1 | TO | JES | East | 180 |
| 2018 | 1 | Flush | ShallowPond | 3 | 0 | 1 | TO | JES | East | 180 |
| 2018 | 1 | Flying | Air | 4 | 0 | 1 | TO | JES | East | 180 |
| 2018 | 1 | Flush | ShallowPond | 4 | 0 | 1 | TO | JES | East | 180 |
| 2018 | 1 | Flying | Air | 2 | 0 | 1 | TO | JES | East | 195 |
| 2018 | 1 | Ground | ShallowPond | 3 | 0 | 1 | TO | JES | East | 210 |
| 2018 | 1 | Ground | ShallowPond | 1 | 0 | 1 | TO | JES | East | 225 |
| 2018 | 1 | Ground | Stream | 4 | 0 | 1 | TO | JES | East | 225 |
| 2018 | 1 | Flush | ShallowPond | 2 | 0 | 1 | JES | TO | East | 225 |
| 2018 | 1 | Flush | DeepPond | 4 | 0 | 1 | JES | TO | East | 225 |
| 2018 | 1 | Ground | ShallowPond | 4 | 0 | 1 | TO | JES | East | 240 |
| 2018 | 1 | Ground | DeepPond | 2 | 0 | 1 | JES | TO | West | 75 |
| 2018 | 1 | Flying | Air | 2 | 0 | 1 | JES | TO | West | 135 |
| 2018 | 1 | Flush | ShallowPond | 1 | 0 | 1 | TO | JES | West | 150 |
| 2018 | 1 | Flush | ShallowPond | 4 | 0 | 1 | TO | JES | West | 165 |
| 2018 | 1 | Flying | Air | NA | 0 | 1 | TO | JES | West | 165 |
| 2018 | 1 | Flush | ShallowPond | 1 | 0 | 1 | JES | TO | West | 165 |
| 2018 | 1 | Ground | ShallowPond | 1 | 0 | 1 | JES | TO | West | 165 |
| 2018 | 1 | Flush | ShallowPond | 3 | 0 | 1 | TO | JES | West | 180 |
| 2018 | 1 | Flush | ShallowPond | 2 | 0 | 1 | JES | TO | West | 210 |
| 2018 | 1 | Flying | Air | 3 | 0 | 1 | JES | TO | West | 210 |
| 2018 | 1 | Flying | Air | 4 | 0 | 1 | JES | TO | West | 210 |
| 2018 | 1 | Ground | ShallowPond | 1 | 0 | 1 | TO | JES | West | 225 |
| 2018 | 1 | Ground | ShallowPond | 2 | 0 | 1 | TO | JES | West | 240 |
| 2018 | 2 | Flying | Air | 2 | 0 | 1 | JES | TO | East | 150 |
| 2018 | 2 | Ground | ShallowPond | 3 | 0 | 1 | TO | JES | West | 240 |
| 2018 | 1 | Flush | ShallowPond | 1 | 1 | 0 | JES | TO | East | 120 |
| 2018 | 1 | Flying | Air | 3 | 1 | 0 | JES | TO | East | 135 |
| 2018 | 1 | Flying | Air | 3 | 1 | 0 | JES | TO | East | 135 |
| 2018 | 1 | Flush | ShallowPond | 2 | 1 | 0 | TO | JES | East | 150 |
| 2018 | 1 | Flush | ShallowPond | 2 | 1 | 0 | JES | TO | East | 150 |
| 2018 | 1 | Flying | Air | 3 | 1 | 0 | JES | TO | East | 150 |
| 2018 | 1 | Flush | ShallowPond | 4 | 1 | 0 | JES | TO | East | 150 |
| 2018 | 1 | Flush | ShallowPond | 2 | 1 | 0 | TO | JES | East | 180 |
| 2018 | 1 | Flush | ShallowPond | 3 | 1 | 0 | TO | JES | East | 180 |
| 2018 | 1 | Flying | Air | 4 | 1 | 0 | TO | JES | East | 180 |
| 2018 | 1 | Flying | Air | 3 | 1 | 0 | TO | JES | East | 195 |
| 2018 | 1 | Flying | Air | 3 | 1 | 0 | TO | JES | East | 195 |
| 2018 | 1 | Flying | Air | 4 | 1 | 0 | TO | JES | East | 210 |
| 2018 | 1 | Flush | ShallowPond | 2 | 1 | 0 | TO | JES | East | 240 |
| 2018 | 1 | Ground | ShallowPond | 1 | 1 | 0 | TO | JES | West | 105 |
| 2018 | 1 | Flying | Air | 4 | 1 | 0 | TO | JES | West | 120 |
| 2018 | 1 | Flush | ShallowPond | 2 | 1 | 0 | JES | TO | West | 135 |
| 2018 | 1 | Ground | ShallowPond | 1 | 1 | 0 | TO | JES | West | 150 |
| 2018 | 1 | Flying | Air | 4 | 1 | 0 | TO | JES | West | 150 |
| 2018 | 1 | Flush | DeepPond | 4 | 1 | 0 | TO | JES | West | 150 |
| 2018 | 1 | Ground | ShallowPond | 3 | 1 | 0 | JES | TO | West | 150 |
| 2018 | 1 | Flush | DeepPond | 3 | 1 | 0 | TO | JES | West | 165 |
| 2018 | 1 | Ground | ShallowPond | 2 | 1 | 0 | JES | TO | West | 165 |
| 2018 | 1 | Ground | ShallowPond | 3 | 1 | 0 | JES | TO | West | 180 |
| 2018 | 1 | Ground | ShallowPond | 1 | 1 | 0 | TO | JES | West | 195 |
| 2018 | 1 | Flying | Air | 4 | 1 | 0 | TO | JES | West | 195 |
| 2018 | 1 | Flying | Air | 4 | 1 | 0 | TO | JES | West | 195 |
| 2018 | 1 | Flying | Air | 4 | 1 | 0 | JES | TO | West | 210 |
| 2018 | 1 | Flying | Air | 3 | 1 | 0 | TO | JES | West | 240 |
| 2018 | 1 | Flying | Air | 4 | 1 | 0 | TO | JES | West | 240 |
| 2018 | 2 | Flush | ShallowPond | 1 | 1 | 0 | TO | JES | East | 225 |
| 2018 | 2 | Flying | Air | 4 | 1 | 0 | TO | JES | West | 195 |
| 2018 | 2 | Flying | Air | 4 | 1 | 0 | JES | TO | West | 210 |
| 2018 | 1 | Flying | Air | 4 | 1 | 1 | JES | TO | East | 120 |
| 2018 | 1 | Flying | Air | 1 | 1 | 1 | TO | JES | East | 135 |
| 2018 | 1 | Flush | ShallowPond | 2 | 1 | 1 | JES | TO | East | 135 |
| 2018 | 1 | Flush | ShallowPond | 2 | 1 | 1 | JES | TO | East | 135 |
| 2018 | 1 | Flying | Air | 3 | 1 | 1 | TO | JES | East | 150 |
| 2018 | 1 | Flying | Air | 3 | 1 | 1 | TO | JES | East | 150 |
| 2018 | 1 | Flying | Air | 1 | 1 | 1 | JES | TO | East | 150 |
| 2018 | 1 | Flying | Air | 1 | 1 | 1 | TO | JES | East | 150 |
| 2018 | 1 | Flying | Air | 1 | 1 | 1 | TO | JES | East | 150 |
| 2018 | 1 | Flying | Air | 2 | 1 | 1 | TO | JES | East | 150 |
| 2018 | 1 | Flush | ShallowPond | 2 | 1 | 1 | JES | TO | East | 150 |
| 2018 | 1 | Ground | ShallowPond | 2 | 1 | 1 | TO | JES | East | 150 |
| 2018 | 1 | Flying | Air | 1 | 1 | 1 | TO | JES | East | 165 |
| 2018 | 1 | Flying | Air | 2 | 1 | 1 | TO | JES | East | 165 |
| 2018 | 1 | Flying | Air | 1 | 1 | 1 | TO | JES | East | 180 |
| 2018 | 1 | Flying | Air | 2 | 1 | 1 | TO | JES | East | 180 |
| 2018 | 1 | Flying | Air | 2 | 1 | 1 | JES | TO | East | 180 |
| 2018 | 1 | Flush | ShallowPond | 2 | 1 | 1 | TO | JES | East | 180 |
| 2018 | 1 | Flush | ShallowPond | 1 | 1 | 1 | TO | JES | East | 195 |
| 2018 | 1 | Flying | Air | 2 | 1 | 1 | JES | TO | East | 210 |
| 2018 | 1 | Flying | Air | 3 | 1 | 1 | JES | TO | East | 210 |
| 2018 | 1 | Flush | ShallowPond | 2 | 1 | 1 | JES | TO | East | 210 |
| 2018 | 1 | Flying | Air | 2 | 1 | 1 | TO | JES | East | 210 |
| 2018 | 1 | Flying | Air | 3 | 1 | 1 | TO | JES | East | 210 |
| 2018 | 1 | Flying | Air | 1 | 1 | 1 | TO | JES | East | 225 |
| 2018 | 1 | Flying | Air | 1 | 1 | 1 | TO | JES | East | 225 |
| 2018 | 1 | Flush | DeepPond | 1 | 1 | 1 | TO | JES | East | 225 |
| 2018 | 1 | Flush | ShallowPond | 1 | 1 | 1 | JES | TO | East | 225 |
| 2018 | 1 | Flying | Air | 3 | 1 | 1 | JES | TO | East | 240 |
| 2018 | 1 | Flush | ShallowPond | 3 | 1 | 1 | TO | JES | East | 240 |
| 2018 | 1 | Flying | Air | 3 | 1 | 1 | TO | JES | East | 240 |
| 2018 | 1 | Flying | Air | 3 | 1 | 1 | TO | JES | East | 240 |
| 2018 | 1 | Flying | Air | 4 | 1 | 1 | JES | TO | East | 255 |
| 2018 | 1 | Flush | DeepPond | 3 | 1 | 1 | JES | TO | East | 255 |
| 2018 | 1 | Flush | ShallowPond | 1 | 1 | 1 | JES | TO | West | 75 |
| 2018 | 1 | Ground | Stream | 1 | 1 | 1 | TO | JES | West | 105 |
| 2018 | 1 | Flying | Air | 1 | 1 | 1 | TO | JES | West | 105 |
| 2018 | 1 | Flying | Air | 1 | 1 | 1 | JES | TO | West | 120 |
| 2018 | 1 | Flying | Air | 1 | 1 | 1 | TO | JES | West | 120 |
| 2018 | 1 | Ground | ShallowPond | 1 | 1 | 1 | JES | TO | West | 120 |
| 2018 | 1 | Flush | ShallowPond | 2 | 1 | 1 | JES | TO | West | 120 |
| 2018 | 1 | Ground | ShallowPond | 2 | 1 | 1 | JES | TO | West | 135 |
| 2018 | 1 | Flush | ShallowPond | 2 | 1 | 1 | JES | TO | West | 150 |
| 2018 | 1 | Flush | ShallowPond | 1 | 1 | 1 | TO | JES | West | 150 |
| 2018 | 1 | Flush | ShallowPond | 2 | 1 | 1 | TO | JES | West | 150 |
| 2018 | 1 | Ground | ShallowPond | 3 | 1 | 1 | JES | TO | West | 150 |
| 2018 | 1 | Flying | Air | 1 | 1 | 1 | JES | TO | West | 165 |
| 2018 | 1 | Flush | ShallowPond | 2 | 1 | 1 | JES | TO | West | 180 |
| 2018 | 1 | Flush | ShallowPond | 1 | 1 | 1 | JES | TO | West | 180 |
| 2018 | 1 | Flush | ShallowPond | 1 | 1 | 1 | TO | JES | West | 180 |
| 2018 | 1 | Ground | ShallowPond | 1 | 1 | 1 | TO | JES | West | 180 |
| 2018 | 1 | Flying | Air | 2 | 1 | 1 | TO | JES | West | 180 |
| 2018 | 1 | Flying | Air | 3 | 1 | 1 | JES | TO | West | 180 |
| 2018 | 1 | Flying | Air | 2 | 1 | 1 | TO | JES | West | 195 |
| 2018 | 1 | Ground | ShallowPond | 2 | 1 | 1 | TO | JES | West | 195 |
| 2018 | 1 | Flying | Air | 3 | 1 | 1 | TO | JES | West | 195 |
| 2018 | 1 | Flying | Air | 3 | 1 | 1 | TO | JES | West | 195 |
| 2018 | 1 | Flush | ShallowPond | 1 | 1 | 1 | JES | TO | West | 210 |
| 2018 | 1 | Ground | ShallowPond | 2 | 1 | 1 | JES | TO | West | 210 |
| 2018 | 1 | Flying | Air | 2 | 1 | 1 | TO | JES | West | 225 |
| 2018 | 1 | Flying | Air | 2 | 1 | 1 | TO | JES | West | 240 |
| 2018 | 1 | Flying | Air | 2 | 1 | 1 | TO | JES | West | 240 |
| 2018 | 1 | Flying | Air | 4 | 1 | 1 | JES | TO | West | 240 |
| 2018 | 2 | Flying | Air | 2 | 1 | 1 | JES | TO | East | 150 |
| 2018 | 2 | Flying | Air | 2 | 1 | 1 | TO | JES | East | 210 |
| 2018 | 2 | Flying | Air | 2 | 1 | 1 | JES | TO | East | 240 |
| 2018 | 2 | Flying | Air | 2 | 1 | 1 | TO | JES | West | 165 |
| 2018 | 2 | Flush | ShallowPond | 1 | 1 | 1 | TO | JES | West | 195 |
| 2018 | 2 | Flush | ShallowPond | 1 | 1 | 1 | JES | TO | West | 210 |
| 2018 | 3 | Flying | Air | 3 | 1 | 1 | TO | JES | East | 135 |
| 2018 | 3 | Ground | ShallowPond | 1 | 1 | 1 | JES | TO | West | 165 |
| 2018 | 3 | Ground | ShallowPond | 2 | 1 | 1 | TO | JES | West | 165 |

**Appendix C. Double-observer (DO) aerial survey king eider data, 2017 and 2018.** Males = number of males observed in the group. Behavior = Flying, Flush, or Ground (neither flying nor flushing). Habitat = 5 types (air, shallow pond, deep pond, ground, or stream). Distance = 4 bins; 1 = 0-50m, 2 = 51-100m, 3 = 101-150m, 4 = 151-200m. Front = observation from front seat (1 = yes, 0 = no). Rear = observation from rear seat (1 = yes, 0 = no). FrontObs, RearObs = identity of front and rear surveyors. TransDir = transect-level variable, direction of the transect. SunDegree = transect-level variable, with due North as 0, the predominate direction from which the sun was hitting the airplane during the transect.

| Year | Males | Behavior | Habitat | Distance | Front | Rear | FrontObs | RearObs | TransDir | SunDegree |
| --- | --- | --- | --- | --- | --- | --- | --- | --- | --- | --- |
| 2017 | 1 | Ground | ShallowPond | 1 | 0 | 1 | JES | TO | East | 150 |
| 2017 | 1 | Ground | ShallowPond | 2 | 0 | 1 | TO | JES | East | 150 |
| 2017 | 1 | Flying | Air | 4 | 0 | 1 | TO | JES | East | 150 |
| 2017 | 1 | Ground | ShallowPond | 1 | 0 | 1 | JES | TO | East | 165 |
| 2017 | 1 | Ground | DeepPond | 2 | 0 | 1 | TO | JES | East | 210 |
| 2017 | 1 | Flying | Stream | 3 | 0 | 1 | TO | JES | East | 210 |
| 2017 | 1 | Ground | ShallowPond | 1 | 0 | 1 | TO | JES | East | 225 |
| 2017 | 1 | Flush | ShallowPond | 1 | 0 | 1 | JES | TO | East | 225 |
| 2017 | 1 | Flying | Air | 3 | 0 | 1 | JES | TO | East | 225 |
| 2017 | 1 | Flying | Ground | 3 | 0 | 1 | TO | JES | East | 225 |
| 2017 | 1 | Ground | ShallowPond | 3 | 0 | 1 | TO | JES | East | 255 |
| 2017 | 1 | Ground | ShallowPond | 3 | 0 | 1 | TO | JES | East | 270 |
| 2017 | 1 | Flush | ShallowPond | 3 | 0 | 1 | JES | TO | West | 150 |
| 2017 | 1 | Flush | ShallowPond | 3 | 0 | 1 | JES | TO | West | 150 |
| 2017 | 1 | Flying | Air | 4 | 0 | 1 | JES | TO | West | 150 |
| 2017 | 2 | Ground | Stream | 4 | 0 | 1 | JES | TO | West | 165 |
| 2017 | 1 | Flying | Air | 3 | 0 | 1 | JES | TO | West | 180 |
| 2017 | 1 | Flush | ShallowPond | 3 | 0 | 1 | JES | TO | West | 210 |
| 2017 | 1 | Flying | Air | 3 | 0 | 1 | JES | TO | West | 225 |
| 2017 | 1 | Flying | Ground | 3 | 0 | 1 | TO | JES | West | 225 |
| 2017 | 1 | Flying | Air | 4 | 0 | 1 | TO | JES | West | 225 |
| 2017 | 1 | Ground | ShallowPond | 1 | 0 | 1 | JES | TO | West | 240 |
| 2017 | 1 | Ground | DeepPond | 3 | 0 | 1 | JES | TO | West | 240 |
| 2017 | 1 | Ground | ShallowPond | 2 | 0 | 1 | JES | TO | West | 255 |
| 2017 | 1 | Flying | Air | 3 | 0 | 1 | TO | JES | West | 285 |
| 2017 | 1 | Ground | ShallowPond | 4 | 1 | 0 | TO | JES | East | 150 |
| 2017 | 1 | Flying | Air | 1 | 1 | 0 | TO | JES | East | 195 |
| 2017 | 2 | Flying | Air | 2 | 1 | 0 | TO | JES | East | 210 |
| 2017 | 1 | Flying | Air | 3 | 1 | 0 | TO | JES | East | 210 |
| 2017 | 1 | Ground | ShallowPond | 2 | 1 | 0 | JES | TO | East | 225 |
| 2017 | 1 | Flying | DeepPond | 3 | 1 | 0 | JES | TO | East | 225 |
| 2017 | 2 | Flying | ShallowPond | 3 | 1 | 0 | JES | TO | East | 225 |
| 2017 | 1 | Ground | ShallowPond | 3 | 1 | 0 | JES | TO | East | 255 |
| 2017 | 1 | Ground | ShallowPond | 2 | 1 | 0 | JES | TO | West | 120 |
| 2017 | 1 | Flying | DeepPond | 2 | 1 | 0 | JES | TO | West | 135 |
| 2017 | 1 | Flying | Air | 3 | 1 | 0 | JES | TO | West | 150 |
| 2017 | 1 | Ground | ShallowPond | 1 | 1 | 0 | TO | JES | West | 165 |
| 2017 | 1 | Ground | DeepPond | 2 | 1 | 0 | TO | JES | West | 165 |
| 2017 | 1 | Flying | Air | 4 | 1 | 0 | JES | TO | West | 165 |
| 2017 | 1 | Flying | DeepPond | 1 | 1 | 0 | JES | TO | West | 180 |
| 2017 | 1 | Ground | DeepPond | 3 | 1 | 0 | JES | TO | West | 195 |
| 2017 | 1 | Ground | ShallowPond | 1 | 1 | 0 | JES | TO | West | 270 |
| 2017 | 2 | Ground | ShallowPond | 1 | 1 | 1 | JES | TO | East | 135 |
| 2017 | 1 | Flying | Air | 3 | 1 | 1 | JES | TO | East | 135 |
| 2017 | 1 | Flush | ShallowPond | 4 | 1 | 1 | JES | TO | East | 135 |
| 2017 | 1 | Flush | ShallowPond | 1 | 1 | 1 | JES | TO | East | 150 |
| 2017 | 1 | Flying | Air | 2 | 1 | 1 | JES | TO | East | 150 |
| 2017 | 1 | Flying | Air | 2 | 1 | 1 | TO | JES | East | 165 |
| 2017 | 1 | Flying | Air | 2 | 1 | 1 | TO | JES | East | 165 |
| 2017 | 1 | Flying | Air | 3 | 1 | 1 | TO | JES | East | 165 |
| 2017 | 1 | Flush | ShallowPond | 2 | 1 | 1 | TO | JES | East | 195 |
| 2017 | 1 | Flying | Air | 3 | 1 | 1 | TO | JES | East | 195 |
| 2017 | 1 | Flying | Air | 1 | 1 | 1 | JES | TO | East | 210 |
| 2017 | 1 | Flying | Air | 1 | 1 | 1 | JES | TO | East | 210 |
| 2017 | 1 | Flush | ShallowPond | 2 | 1 | 1 | TO | JES | East | 210 |
| 2017 | 1 | Flush | ShallowPond | 2 | 1 | 1 | JES | TO | East | 210 |
| 2017 | 1 | Flush | ShallowPond | 4 | 1 | 1 | TO | JES | East | 210 |
| 2017 | 1 | Ground | ShallowPond | 1 | 1 | 1 | JES | TO | East | 225 |
| 2017 | 1 | Flush | ShallowPond | 2 | 1 | 1 | JES | TO | East | 225 |
| 2017 | 1 | Flush | ShallowPond | 2 | 1 | 1 | JES | TO | East | 225 |
| 2017 | 1 | Ground | Stream | 2 | 1 | 1 | TO | JES | East | 225 |
| 2017 | 1 | Ground | ShallowPond | 1 | 1 | 1 | JES | TO | East | 255 |
| 2017 | 1 | Ground | DeepPond | 3 | 1 | 1 | TO | JES | East | 270 |
| 2017 | 1 | Flush | ShallowPond | 2 | 1 | 1 | TO | JES | East | 285 |
| 2017 | 1 | Flush | ShallowPond | 1 | 1 | 1 | JES | TO | West | 135 |
| 2017 | 1 | Flying | Air | 1 | 1 | 1 | TO | JES | West | 150 |
| 2017 | 1 | Ground | ShallowPond | 1 | 1 | 1 | TO | JES | West | 165 |
| 2017 | 1 | Ground | Stream | 1 | 1 | 1 | JES | TO | West | 165 |
| 2017 | 1 | Ground | ShallowPond | 1 | 1 | 1 | JES | TO | West | 210 |
| 2017 | 1 | Ground | DeepPond | 2 | 1 | 1 | JES | TO | West | 210 |
| 2017 | 1 | Flying | Air | 4 | 1 | 1 | JES | TO | West | 210 |
| 2017 | 1 | Ground | DeepPond | 1 | 1 | 1 | JES | TO | West | 225 |
| 2017 | 1 | Flush | ShallowPond | 1 | 1 | 1 | TO | JES | West | 225 |
| 2017 | 2 | Flying | Air | 2 | 1 | 1 | JES | TO | West | 225 |
| 2017 | 2 | Flying | Air | 3 | 1 | 1 | TO | JES | West | 225 |
| 2017 | 1 | Flying | Air | 3 | 1 | 1 | TO | JES | West | 225 |
| 2017 | 2 | Ground | ShallowPond | 1 | 1 | 1 | JES | TO | West | 240 |
| 2017 | 1 | Flying | Air | 2 | 1 | 1 | JES | TO | West | 240 |
| 2017 | 1 | Ground | DeepPond | 2 | 1 | 1 | JES | TO | West | 240 |
| 2017 | 3 | Flying | Air | 1 | 1 | 1 | TO | JES | West | 255 |
| 2017 | 1 | Flying | Air | 3 | 1 | 1 | TO | JES | West | 255 |
| 2017 | 1 | Flying | Air | 3 | 1 | 1 | TO | JES | West | 255 |
| 2017 | 1 | Flush | ShallowPond | 1 | 1 | 1 | JES | TO | West | 270 |
| 2017 | 1 | Ground | ShallowPond | 1 | 1 | 1 | JES | TO | West | 270 |
| 2017 | 1 | Flush | Stream | 1 | 1 | 1 | JES | TO | West | 270 |
| 2017 | 1 | Ground | ShallowPond | 2 | 1 | 1 | JES | TO | West | 270 |
| 2017 | 1 | Flush | ShallowPond | 1 | 1 | 1 | TO | JES | West | 285 |
| 2017 | 1 | Flying | Air | 2 | 1 | 1 | TO | JES | West | 285 |
| 2018 | 1 | Flush | ShallowPond | 3 | 0 | 1 | JES | TO | East | 135 |
| 2018 | 1 | Flush | ShallowPond | 4 | 0 | 1 | JES | TO | West | 135 |
| 2018 | 2 | Flush | DeepPond | 3 | 0 | 1 | JES | TO | West | 120 |
| 2018 | 1 | Flying | Air | 3 | 0 | 1 | TO | JES | East | 150 |
| 2018 | 1 | Flying | Air | 4 | 0 | 1 | TO | JES | East | 150 |
| 2018 | 1 | Flying | Air | 1 | 0 | 1 | JES | TO | East | 150 |
| 2018 | 1 | Flying | Air | 4 | 0 | 1 | JES | TO | East | 150 |
| 2018 | 1 | Flying | Air | 3 | 0 | 1 | TO | JES | East | 180 |
| 2018 | 1 | Flying | Air | 4 | 0 | 1 | TO | JES | East | 180 |
| 2018 | 1 | Flying | Air | 3 | 0 | 1 | JES | TO | East | 180 |
| 2018 | 1 | Flying | Air | 3 | 0 | 1 | TO | JES | East | 195 |
| 2018 | 1 | Flying | Air | 2 | 0 | 1 | JES | TO | East | 225 |
| 2018 | 1 | Flying | Air | 1 | 0 | 1 | JES | TO | East | 240 |
| 2018 | 1 | Flying | Air | 4 | 0 | 1 | TO | JES | West | 225 |
| 2018 | 1 | Flying | Air | 1 | 0 | 1 | JES | TO | West | 225 |
| 2018 | 2 | Flying | Air | 2 | 0 | 1 | TO | JES | East | 195 |
| 2018 | 2 | Flying | Air | 3 | 0 | 1 | TO | JES | West | 180 |
| 2018 | 2 | Flying | Air | 4 | 0 | 1 | TO | JES | West | 195 |
| 2018 | 1 | Ground | ShallowPond | 1 | 0 | 1 | JES | TO | East | 135 |
| 2018 | 1 | Ground | ShallowPond | 1 | 0 | 1 | JES | TO | East | 135 |
| 2018 | 1 | Ground | ShallowPond | 1 | 0 | 1 | JES | TO | East | 135 |
| 2018 | 1 | Ground | ShallowPond | 4 | 0 | 1 | JES | TO | East | 180 |
| 2018 | 1 | Ground | ShallowPond | 2 | 0 | 1 | TO | JES | West | 105 |
| 2018 | 1 | Ground | ShallowPond | 4 | 0 | 1 | JES | TO | West | 135 |
| 2018 | 1 | Ground | ShallowPond | 1 | 0 | 1 | JES | TO | West | 150 |
| 2018 | 1 | Ground | ShallowPond | 1 | 0 | 1 | TO | JES | West | 165 |
| 2018 | 1 | Ground | ShallowPond | 1 | 0 | 1 | TO | JES | West | 180 |
| 2018 | 2 | Ground | ShallowPond | 3 | 0 | 1 | JES | TO | West | 135 |
| 2018 | 3 | Ground | DeepPond | 3 | 0 | 1 | TO | JES | East | 225 |
| 2018 | 1 | Flush | ShallowPond | 2 | 1 | 0 | TO | JES | East | 135 |
| 2018 | 1 | Flush | Stream | 1 | 1 | 0 | TO | JES | East | 150 |
| 2018 | 1 | Flush | ShallowPond | 2 | 1 | 0 | TO | JES | East | 165 |
| 2018 | 1 | Flush | ShallowPond | 3 | 1 | 0 | TO | JES | East | 180 |
| 2018 | 1 | Flush | ShallowPond | 2 | 1 | 0 | TO | JES | East | 240 |
| 2018 | 1 | Flush | ShallowPond | 4 | 1 | 0 | JES | TO | East | 255 |
| 2018 | 1 | Flush | ShallowPond | 1 | 1 | 0 | TO | JES | West | 135 |
| 2018 | 1 | Flush | Stream | 1 | 1 | 0 | JES | TO | West | 135 |
| 2018 | 1 | Flush | ShallowPond | 1 | 1 | 0 | TO | JES | West | 180 |
| 2018 | 1 | Flush | ShallowPond | 2 | 1 | 0 | TO | JES | West | 180 |
| 2018 | 1 | Flush | ShallowPond | 3 | 1 | 0 | JES | TO | West | 225 |
| 2018 | 1 | Flush | ShallowPond | 1 | 1 | 0 | JES | TO | West | 240 |
| 2018 | 1 | Flying | Air | 3 | 1 | 0 | JES | TO | East | 135 |
| 2018 | 1 | Flying | Air | 2 | 1 | 0 | TO | JES | East | 150 |
| 2018 | 1 | Flying | Air | 2 | 1 | 0 | TO | JES | East | 195 |
| 2018 | 1 | Flying | Air | 4 | 1 | 0 | TO | JES | West | 135 |
| 2018 | 1 | Flying | Air | 4 | 1 | 0 | JES | TO | West | 150 |
| 2018 | 2 | Flying | Air | 4 | 1 | 0 | TO | JES | West | 165 |
| 2018 | 1 | Ground | ShallowPond | 1 | 1 | 0 | JES | TO | West | 180 |
| 2018 | 1 | Ground | ShallowPond | 1 | 1 | 0 | JES | TO | West | 210 |
| 2018 | 1 | Ground | Stream | 1 | 1 | 0 | TO | JES | West | 225 |
| 2018 | 2 | Ground | ShallowPond | 1 | 1 | 0 | TO | JES | West | 105 |
| 2018 | 1 | Flush | ShallowPond | 3 | 1 | 1 | TO | JES | East | 180 |
| 2018 | 1 | Flush | ShallowPond | 1 | 1 | 1 | JES | TO | West | 75 |
| 2018 | 1 | Flush | ShallowPond | 3 | 1 | 1 | TO | JES | West | 150 |
| 2018 | 1 | Flush | ShallowPond | 1 | 1 | 1 | TO | JES | West | 195 |
| 2018 | 1 | Flush | ShallowPond | 3 | 1 | 1 | JES | TO | West | 225 |
| 2018 | 1 | Flying | Air | 3 | 1 | 1 | JES | TO | East | 120 |
| 2018 | 1 | Flying | Air | 4 | 1 | 1 | JES | TO | East | 120 |
| 2018 | 1 | Flying | Air | 1 | 1 | 1 | TO | JES | East | 150 |
| 2018 | 1 | Flying | Air | 2 | 1 | 1 | TO | JES | East | 150 |
| 2018 | 1 | Flying | Air | 3 | 1 | 1 | TO | JES | East | 150 |
| 2018 | 1 | Flying | Air | 3 | 1 | 1 | TO | JES | East | 150 |
| 2018 | 1 | Flying | Air | 1 | 1 | 1 | TO | JES | East | 165 |
| 2018 | 1 | Flying | Air | 2 | 1 | 1 | JES | TO | East | 165 |
| 2018 | 1 | Flying | Air | 2 | 1 | 1 | TO | JES | East | 165 |
| 2018 | 1 | Flying | Air | 1 | 1 | 1 | TO | JES | East | 180 |
| 2018 | 1 | Flying | Air | 1 | 1 | 1 | JES | TO | East | 180 |
| 2018 | 1 | Flying | Air | 3 | 1 | 1 | TO | JES | East | 180 |
| 2018 | 1 | Flying | Air | 3 | 1 | 1 | TO | JES | East | 180 |
| 2018 | 1 | Flying | Air | 4 | 1 | 1 | TO | JES | East | 180 |
| 2018 | 1 | Flying | Air | 2 | 1 | 1 | TO | JES | East | 195 |
| 2018 | 1 | Flying | Air | 1 | 1 | 1 | JES | TO | East | 210 |
| 2018 | 1 | Flying | Air | 1 | 1 | 1 | TO | JES | East | 210 |
| 2018 | 1 | Flying | Air | 2 | 1 | 1 | TO | JES | East | 225 |
| 2018 | 1 | Flying | Air | 3 | 1 | 1 | JES | TO | East | 255 |
| 2018 | 1 | Flying | Air | 4 | 1 | 1 | JES | TO | East | 255 |
| 2018 | 1 | Flying | Air | 3 | 1 | 1 | JES | TO | West | 75 |
| 2018 | 1 | Flying | Air | 1 | 1 | 1 | TO | JES | West | 135 |
| 2018 | 1 | Flying | Air | 2 | 1 | 1 | JES | TO | West | 135 |
| 2018 | 1 | Flying | Air | 2 | 1 | 1 | TO | JES | West | 135 |
| 2018 | 1 | Flying | Air | 3 | 1 | 1 | TO | JES | West | 135 |
| 2018 | 1 | Flying | Air | 2 | 1 | 1 | JES | TO | West | 165 |
| 2018 | 1 | Flying | Air | 1 | 1 | 1 | JES | TO | West | 225 |
| 2018 | 1 | Flying | Air | 1 | 1 | 1 | JES | TO | West | 240 |
| 2018 | 1 | Flying | Air | 2 | 1 | 1 | TO | JES | West | 240 |
| 2018 | 1 | Flying | Air | 2 | 1 | 1 | TO | JES | West | 240 |
| 2018 | 1 | Flying | Air | 3 | 1 | 1 | JES | TO | West | 240 |
| 2018 | 1 | Flying | Air | 4 | 1 | 1 | JES | TO | West | 240 |
| 2018 | 2 | Flying | Air | 3 | 1 | 1 | TO | JES | East | 180 |
| 2018 | 2 | Flying | Air | 3 | 1 | 1 | TO | JES | West | 180 |
| 2018 | 5 | Flying | Air | 4 | 1 | 1 | JES | TO | West | 240 |
| 2018 | 1 | Ground | ShallowPond | 2 | 1 | 1 | TO | JES | West | 105 |
| 2018 | 1 | Ground | ShallowPond | 2 | 1 | 1 | TO | JES | West | 135 |
| 2018 | 1 | Ground | Stream | 2 | 1 | 1 | JES | TO | West | 135 |
| 2018 | 1 | Ground | ShallowPond | 2 | 1 | 1 | JES | TO | West | 135 |
| 2018 | 1 | Ground | ShallowPond | 3 | 1 | 1 | JES | TO | West | 210 |
| 2018 | 1 | Ground | ShallowPond | 3 | 1 | 1 | TO | JES | West | 240 |
| 2018 | 2 | Ground | DeepPond | 2 | 1 | 1 | TO | JES | West | 180 |
