# Supplementary material for: Periodicity in Steller’s eider (Polysticta stelleri) population size and density on the Arctic Coastal Plain, Alaska, revealed using generalized additive models
Source: PLoS One. 2026 Apr 30;21(4):e0347122. doi: 10.1371/journal.pone.0347122 (PMC13132232; doi:10.1371/journal.pone.0347122)
Supplement: Attachment — Submitted filename: Response to Reviewers.docx [file pone.0347122.s002.docx]

Response to Reviewers

Dear Editor,

Thank you and thanks to the reviewers for providing the reviews and formatting guidance. I have revised the manuscript as suggested. I have also revised all figures by (1) making all multi-panel figures one image and (2) re-formatting each figure as a tiff file. This significantly changed Fig 15, which now shows more regions of the spectrogram. Just to be clear, I have not changed any analysis for Fig 15, only the file format. In the original image in eps format, the shaded region could not be displayed, I believe because this region is a separate “raster layer” and eps format can only show one layer. The new figure is much better. In terms of other review comments, I have accepted all of them and made the needed changes.

In addition to the typos and revisions suggested by the reviewers, I have also found a few more typos and made a few other small revisions, as detailed in the track changes version of the PDF.

Below I respond to your comment under “Journal Requirements” and then respond to each comment of the reviewers, with my response in *italics*.

Thank you for the opportunity to revise this manuscript and for your efforts.

Erik

Comments under “Journal Requirements”:

In Comment #1 about style requirements, I have thoroughly revised the manuscript and accompanying .tex file to adhere to your style requirements. The original manuscript and .tex file was produced using the PLOS Latex extension for Quarto, which is based on your latex template. During this revision, I tried to use your template directly but could not get it to compile. I am not a Latex expert, so I can’t provide any reasons for this, it is probably due to my lack of knowledge. By visual inspection, the revised PDF appears to follow your guidelines. Note that there are some small discrepancies between your provided Latex template and the online “Affiliations guidance”. Please let me know if there is more to do.

In Comment #2 on code sharing, I have made all code used for this report available at <https://github.com/USFWS/STEI-estimates> ,as noted by the reviewers. Upon acceptance of the manuscript, I can do a “code release” on GitHub where the code version for this manuscript is packaged together as a downloadable zip file and associated with the publication.

In Commet#3 you asked for clarification about peer-review and “formal publication” of a pervious report that the current manuscript is based on. The previous report can be found at <https://doi.org/10.7944/3vzp-0r93>. This previous report has never undergone external peer-review, as has the current manuscript, and was only posted to our (USFWS) data repositories catalog (we use the USGS ScienceBase). It is not clear to me what is meant by “formal publication”, but I would consider posting the report to our web-catalog equivalent to posting to a pre-print server, as PLOS encourages authors to do. In addition, the current manuscript has been significantly shortened and revised over the posted report.

In Comment #4 about copyrighted map material (Fig 1 and 2), you noted that I must obtain permission to publish these or use public domain data for maps. Please note that all map data used for my figures are US Government works and are in the Public Domain. Specifically, the survey area polygons were created by us (the US Fish and Wildlife Service) and the outline of Alaska was sourced from the open-source R package spData, which sources the data from the US Census Bureau. I have added the following to the captions of figure 1 and 2:

All map data are products of the U.S. Government and are in the Public Domain.

In Comment #5 on Table 3, I have changed the title of the supporting information to “S4 Table 3”. I revised the text in the manuscript to read “S4 Table” and included a description of S4 Table 3 that clearly states that Table 3 is found within the text of the contents of the supplemental information.

Your Comment #6 is not applicable. The reviewers did not suggest any additional citations.

In Comment #7 on the reference list. There have been no changes during revision. I have not cited any retracted papers. I believe it to be complete.

Response to reviewers (my response is in *italics*)

**Reviewer 1:**

1. L 117. Please replace “the above” by “equation (2)”

*Revised.*

2. L 351. Replace “miss-identification” by “misidentification”

*Revised.*

3. L 360. Delete “and” before “Medium”

*Revised.*

4. L 462. Replace “spectorgram” by “spectrogram”

*Revised.*

5. L 465. Replace “ocilation” bny “oscillation”

*Revised.*

6. L 577. Replace “miss-matched” by “mismatched”

*Revised.*

**Reviewer 2:**

Specific Comments:
• Lines 113-119: The concerns highlighted here may warrant further explanation – it’s not made clear here exactly why the estimator is better than the ratio estimator if there are issues in estimation with a strong density gradient. Given the system and the assumptions of a ratio estimator I agree that this is likely better, but this section could use work to explain why including citations if possible. Additionally, the last sentence includes “case” twice, I would suggest the second one be changed to “specific system”.

*I agree and have revised the paragraph to include the specific reason the ratio estimator fails in this case. I had originally neglected to say that the advantage of the ratio estimator depends on a positive correlation between the two variables (here animal count and transect length/area). In this survey region, most animals are observed in the north on shorter transects (a consequence of the animal distribution, triangle shape of the area, and east-west transects). Therefore, there is a negative correlation between transect length and animal count and the ratio estimator does very poorly (i.e., has high variance) compared to other estimators. Therefore, I use a line transect estimator commonly used in distance sampling (Fewster). Here is the revised first half of the paragraph leading up to Equation (1) and (2):*

*I calculated a design-based estimate using formula R3 of Fewster et al. [24], (also see [25], p.79), modified for strip transects. A ratio estimator, which is more common for strip-transect surveys [26, 27, 28], was investigated but found to be poor (very high variance) because most observations of Steller's eiders are on short transects in the northern end of the triangular-shape area and longer transects in the south rarely have observed eiders. The ratio estimator can provide lower variance estimates when there is a positive correlation between the two variables (here, animal count and transect length or area) [26, 28]. In this survey area, however, there is a negative correlation between count and transect length (or area). This makes the ratio estimator worse than a simple plot-based estimates or the "R3" estimate of Fewster we use here [24].*

• Line 465: typo, “ocilation” ◊ “oscillation”

*Revised.*

• Line 564: “When” seems to start with a bolded “W” – this occurs in a couple of other locations but it may be a rendering problem in adobe.

*Interesting. I see the same effect in the PLOS-formatted version without figures but the effect is not in the supplemental version I submitted that included figures in the text. I have checked the .tex document, and no special formatting is applied to this character. I assume this is an Adobe issue and can be dealt with during copy editing if necessary. During the revision, this same effect appears in the compiled PDF when viewed in Adobe but not in the RStudio PDF viewer, so I assume this is in fact an Adobe issue as the reviewer suggested.*

• Figures
o Figures 1 and 2 show the same area with the same distance but are different sizes – consider constraining the output size to be consistent.

*I have revised this and all other figures. These two figures are now the same size.*

o Figure 3 may be easier to read as a “lollipop” chart and better y axis – but not necessary.

*I agree. The figure is now a “lollipop” chart with a slight offset between the two surveys. Thanks for the suggestion.*

o Figure 4 is the only two-part figure that shows up together and has A/B labels within the plot. Check all of the two-part and three-part figures for consistency before resubmission.

*I have revised this and all figures so that each is one image file with labels created in the figure image.*

o Figure 9B doesn’t seem necessary for communicating any results – consider removing or emphasizing the purpose of including it.

*I agree. Panel B of Figure 9 has been removed. The time series is now displayed only in Figure 10, which is sufficient to show the random effects on the response scale.*

o Figure 15 “period” is really far from the numbers of the y-axis. Might be easier to see what is happening if the x-axis had more years but turn then 45 or 90 degrees.

*I increased the number of year tick marks and labels and re-sized the figure. I was able to add year labels without needing to turn them from the horizontal. I moved the “Period” title a little closer to the y axis. Note that in the new figure in tiff format, areas of the spectrogram outside the “cone of influence” (slightly shaded) are now shown. In the original submitted figure (eps format) this area was dropped, I believe because eps format cannot handle more than one raster layer in an image. For submission, I did not understand this difference between eps and tiff formats, and I believe the revised (tiff) figure is better than the eps version.*

o The trends/abundance figures may benefit from reducing the background noise, specifically, reducing the number of gridlines, making the background white, etc. Just a style choice but it would be easier to read.

*I removed the grey background (ggplot theme_minimal) and the minor grid lines in all figures.*
